# Liposomes and Extracellular Vesicles as Drug Delivery Systems: A Comparison of Composition, Pharmacokinetics, and Functionalization

**DOI:** 10.1002/adhm.202100639

**Published:** 2021-06-24

**Authors:** Luke van der Koog, Timea B. Gandek, Anika Nagelkerke

**Affiliations:** ^1^ Molecular Pharmacology Groningen Research Institute of Pharmacy GRIAC Research Institute, University Medical Center Groningen University of Groningen P.O. Box 196, XB10 Groningen 9700 AD The Netherlands; ^2^ Pharmaceutical Analysis Groningen Research Institute of Pharmacy University of Groningen P.O. Box 196, XB20 Groningen 9700 AD The Netherlands

**Keywords:** biodistribution, cellular uptake, targeting moiety incorporation, therapeutic cargo delivery, vesicle functionalization

## Abstract

Over the past decades, lipid‐based nanoparticle drug delivery systems (DDS) have caught the attention of researchers worldwide, encouraging the field to rapidly develop improved ways for effective drug delivery. One of the most prominent examples is liposomes, which are spherical shaped artificial vesicles composed of lipid bilayers and able to encapsulate both hydrophilic and hydrophobic materials. At the same time, biological nanoparticles naturally secreted by cells, called extracellular vesicles (EVs), have emerged as promising more complex biocompatible DDS. In this review paper, the differences and similarities in the composition of both vesicles are evaluated, and critical mediators that affect their pharmacokinetics are elucidate. Different strategies that have been assessed to tweak the pharmacokinetics of both liposomes and EVs are explored, detailing the effects on circulation time, targeting capacity, and cytoplasmic delivery of therapeutic cargo. Finally, whether a hybrid system, consisting of a combination of only the critical constituents of both vesicles, could offer the best of both worlds is discussed. Through these topics, novel leads for further research are provided and, more importantly, gain insight in what the liposome field and the EV field can learn from each other.

## Graphical abstract

Liposomes are one of the most prominent examples of a drug delivery system. The last decade has seen the arrival of a new kid on the block: Extracellular vesicles. This review compares the use of these lipid‐based nanocarriers. Composition and potential for engineering are compared. Also, differences and similarities in pharmacokinetic properties, such as cellular uptake and biodistribution are highlighted.

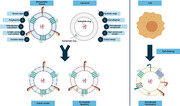

## 1 Introduction

Nanoparticle systems have been perceived as the holy grail for effective drug delivery for decades. The ideal nanoparticle carries the drug‐load safely to a predefined target. There, it is capable of releasing its cargo intracellularly or in the extracellular space where the drug can be directly internalized and exert the desired action. En route, the nanoparticle prevents unwanted interactions of the drug‐load with non‐target tissues and where needed, it will enhance the circulation time of the encapsulated drug and enable sustained release. In this context, liposomes—a class of synthetic lipid nanoparticles—have been explored in depth.^[^ ^1^ ^]^ Liposomes were discovered in the 1960s and are made up of a lipid bilayer, which encloses an aqueous core. Hydrophilic drugs can be included in this core, whereas the lipid bilayer offers space for integration of hydrophobic drugs. Liposomes can significantly alter the pharmacokinetics of drugs. By incorporating compounds in liposomes, they may be protected against dilution and degradation or inactivation in the circulation. However, despite the evident advantages of drug delivery via liposomes, their clinical application has seen substantial biological barriers to be conquered, such as, rapid clearance from the bloodstream, off‐target accumulation in clearance organs, and triggering of the innate immune response.^[^ ^2^ ^]^

Recently, extracellular vesicles (EVs) have emerged as a more complex form of liposomes, but with a biological origin. EVs are nano‐sized vesicles, enveloped by a complex lipid bilayer. They are released from viable cells as exosomes and microvesicles, in a constitutive manner or in response to certain stimuli. Exosomes are generated within multivesicular bodies in the endosomal system. Fusion of the multivesicular body with the plasma membrane, leads to release of the exosomes. Exosomes have a reported size between 30 and 150 nm. Microvesicles on the other hand, have reported sizes between 100 and 1000 nm and are released directly from the plasma membrane.^[^ ^3^ ^]^ Due to their overlap in size and other characteristics, it is currently challenging to isolate pure exosome or microvesicle populations. Therefore, in this review, we will refer to exosomes and microvesicles with the more generic term EVs, as was recommended in the International Society for Extracellular Vesicle guidelines.^[^ ^4^ ^]^ Similarly to liposomes, numerous studies to date have reported the incorporation of drugs into EVs, to assess their suitability as drug delivery entities, as was extensively reviewed by Elsharkasy and coworkers, among others.^[^ ^5^ ^]^ Additionally, EVs offer extensive opportunity for further engineering, for example to optimize their capacity as drug delivery vehicles.^[^ ^6^ ^]^

In this review paper, we will assess the differences and similarities between liposomes and EVs in the context of drug delivery. We will explore the critical composition of liposomes and EVs, and zoom in on important mediators, such as, lipids and proteins, which could directly or indirectly contribute to their pharmacokinetics. We will elaborate on the engineering potential of both liposomes and EVs to enhance favorable pharmacokinetic characteristics in order for these vesicles to function as effective drug delivery systems (DDS). In addition, we will explore the use of EVs for the delivery of small molecules and nucleic acids and make a comparison to liposomes. Key in this discussion is what the EV and liposome field could learn from one another and whether a hybrid system using only the critical constituents, could offer the best of both worlds.

## 2 Lipid composition of Liposomes and Extracellular Vesicles

### 2.1 Lipid Composition of Liposomes Influences Physicochemical Properties

Liposomes with a diameter of 70 nm are estimated to consist of around 60 000 phospholipid molecules.^[^ ^7^ ^]^ This offers huge potential for extensive tailoring of their lipid composition, especially with the vast choice of lipids to select from (see Table S1, Supporting Information, for an overview). A large range of different head groups, chain lengths and chain saturations is available, both from synthetic, as well as, natural sources. The head groups of the various lipid molecules differ in the charge they carry. Cationic lipids carry a net positive charge and as such induce a positive charge on the liposome surface. Liposomes made from this type of lipid can facilitate electrostatic interactions with negatively charged DNA and the cell membrane. Anionic lipids carry a negative charge, and are more alike to the cell membrane and the membrane of EVs. Zwitterionic lipids contain both positive and negative charges, resulting in a net charge that is neutral.

**Figure 1:**
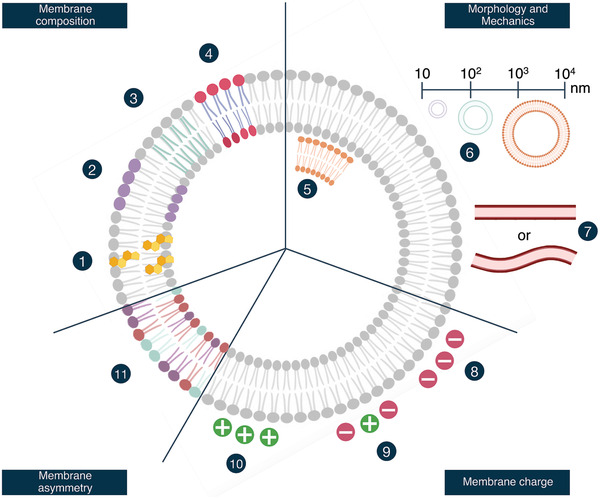
Overview of key compositional parameters of liposomes and EVs. Liposomes typically contain increasing levels of 1) cholesterol to improve vesicle stability and also in EVs high levels of cholesterol have been reported. Liposomes can be made from various lipids, with different 2) head groups, 3) chain lengths, and 4) chain saturation. This also holds true for EVs, which have shown highly complex lipid compositions. Together with cholesterol content, the characteristics of the lipids present are key parameters for pharmacokinetic properties. 5) Lamellarity and 6) size depend on the method that is used for the production of liposomes. 7) Fluidity and mechanics of the membrane are dependent on the mixture of the different lipids and cholesterol that is being used. In EVs, only limited control over (5–7) is present. 8–10) The net charge of the vesicles is dependent on the mixture of the different lipids and cholesterol that is being used. EVs typically carry a net negative charge, whereas for liposomes this parameter can be controlled. 11) EVs potentially have an asymmetry in their lipid bilayers. Incorporation of this feature into liposomes is part of ongoing research.

The extensive choice of lipids allows for the generation of numerous liposomal compositions. With the relatively high curvature of the membrane as a result of the nano‐size, appropriate packing of lipids and therefore bilayer stability is not always guaranteed. Cholesterol is often used as an additive to rigidify the lipid bilayer and to stabilize the liposome structure. Cholesterol molecules can be integrated in the membrane to literally fill in any gaps. In addition, liposome size and lamellarity are parameters that can be controlled. In terms of size, liposomes are categorized as small, large, or giant vesicles. Though the precise classification varies amongst publications, overall small vesicles have a size ranging between 20 and 100 nm, large vesicles between 100 and 1000 nm and giant vesicles are typically larger than 1000 nm.^[^ ^8^ ^]^ Lamellarity reflects the presence of internal lipid structures within a lipid bilayer vesicle. Vesicles can be classified as unilamellar—consisting of a single outer lipid bilayer, as multilamellar—in which consecutive, concentric lipid bilayers are present with in a single outer lipid bilayer, or as multivesicular – where separate, smaller sized vesicles are contained within a single outer lipid bilayer.^[^ ^9^ ^]^ As such, classes of vesicles that are often differentiated are small unilamellar vesicles (SUVs), large unilamellar vesicles (LUVs), giant unilamellar vesicles, and multilamellar vesicles (MLVs). Lamellarity can be a factor of influence in the efficiency with which cargo is encapsulated, but also the subsequent release of cargo and therefore the fate of a drug after uptake in the cell.^[^ ^10^ ^]^ Furthermore, depending on the lipid formulation chosen, fluidity of the lipid bilayer can be tailored. Fluidity is a parameter that is important in the context of drug delivery, as we will explain later on in this review (Section 4.3.). For an overview of liposome preparation methods, we would like to refer to Has and Sunthar.^[^ ^11^ ^]^ It is worth mentioning that standard procedures with which liposomes are manufactured, generates a random mixture of lipids in the lipid bilayers and therefore does not allow for an asymmetrical distribution of lipid species. For a schematic overview of the various compositional parameters that can be controlled in liposomes, we would like to refer to **Figure** **1**.

### 2.2 Extracellular Vesicles Carry a Distinctive Lipidome

Similar to liposomes, EVs are also enclosed by a lipid bilayer. Though it has not been the topic of in‐depth study, it would appear that also EVs can present as multilamellar structures.^[^ ^12^ ^]^ Extensive lipidomic analyses of EVs from various sources have shown that EVs mainly contain lipid species that are also present in the plasma membrane. However, there is substantial difference in the ratios between different lipids in the EV membrane compared to the cell membrane, with EVs showing an enrichment in glycosphingolipids, sphingomyelins, phosphatidylethanolamines, phosphatidylserines, phosphatidylcholines, and cholesterol. An overview of lipidomic analyses is provided in Table S2, Supporting Information. The identity of the lipid head group, the head group charge, length, and saturation of fatty acid tails are important factors contributing to the complexity of the lipid profiles of EVs.^[^ ^13^ ^]^ As EVs are nano‐ to micro‐sized vesicles, the lipid bilayer is also a highly curved structure, similar to liposomes. This is reflected in the enrichment in lipid species that permit the positive curvature in the outer membrane, such as, lipids with one fatty acid chain, as well as, negative curvature in the inner membrane, such as, lipids with more protruding fatty acid chains.^[^ ^13^ ^]^ The lipids present also define the membrane's hydrophobicity and polarity. Similar to liposomes, the lipids found in EVs can be categorized as anionic (such as, glycerophosphatidic acid, glycerophosphoglycerol, glycerophosphoinositol), weakly anionic (ceramide and glycerophosphoethanolamine) or neutral (e.g., mono‐, di‐, and triacylglycerol, cholesterol esters, glycerophosphocholine, sphingomyelin). However, the complex composition of biological membranes with numerous variations in lipid species will determine the overall physicochemical properties of the EVs. Analysis of the *ζ*‐potential for EVs is reported to be negative.^[^ ^14^ ^]^ This may be due to the presence of negatively charged lipids in the EV membrane, such as, phosphatidylserine, but may also be attributed to some extent to the presence of glycan‐moieties.^[^ ^15^ ^]^

Despite evident similarities between EVs derived from different sources, their precise lipid composition depends on the cell type of origin (see Table S2, Supporting Information). In addition to this inter‐vesicle variation, differences between vesicles originating from the same cell type, have also been described. Zhang and colleagues used asymmetric flow field‐flow fractionation to separate subgroups in the EV population.^[^ ^16^ ^]^ Subsequent lipidomic analysis revealed differences in lipid quantity and composition. In most of the subgroups generated, phosphatidylcholine was the major lipid species found, at ≈46–89% of the total lipids. Additional phospholipids, such as, phosphatidylethanolamine and phosphatidylserine, accounted for 2–6% of total lipids identified. Depending on the cell type from which the vesicles were obtained and the subgroup analyzed, between 2% and 28% of the lipid population was made up of sphingomyelin. Cholesterol analysis was not reported in this study. Overall, differences in total lipid content and lipid composition were found, depending on the secreting cell type and vesicle subgroup analyzed.^[^ ^16^ ^]^ Furthermore, a study by Durcin and coworkers used successive differential centrifugation steps to isolate a population of large EVs and a population of small EVs. They showed that the cholesterol content was enhanced in the small EVs, whereas the externalized phosphatidylserine was more abundant in the large EVs.^[^ ^17^ ^]^ Dang et al. examined the difference in lipid composition between EVs released from the apical versus the basolateral side of polarized epithelial cells. For this purpose, they used a mouse cortical collecting duct principal cell line. Lipid composition was shown to differ between EVs released from the apical membrane and basolateral membrane. EVs released from the apical side contained more sphingomyelin than those from the basolateral side. On the other hand, EVs from the basolateral side showed increased levels of cardiolipins, ceramides, and other phospholipids.^[^ ^18^ ^]^ Therefore, cell source, EV subpopulation analyzed and even cell polarity can affect lipid composition of EVs.

The culture conditions of cells have been shown to influence the cellular lipidome. For example, increasing cell density was shown to lead to changes in the lipid composition of HEp‐2 cells. The most profound effects were found on phosphatidic acid, diacylglycerol, lysophosphatidylethanolamine, neutral glycosphingolipids, and cholesteryl esters.^[^ ^19^ ^]^ Furthermore, differences in the composition of the cell culture medium, for example, by the addition of lipid precursors, can influence the lipid composition of cells.^[^ ^20^ ^]^ It is feasible that conditions such as these are also reflected in the lipidome of EVs. Indeed, exposure of prostate cancer cells (PC‐3) to increased levels of ether lipids, in the form of hexadecylglycerol, led to the cells containing higher levels of ether lipids and releasing larger numbers of EVs. The released EVs contained enhanced levels of ether lipids, especially ether‐linked phosphatidylethanolamine, whilst they had reduced levels of phosphatidylserine.^[^ ^21^ ^]^ In addition, supplementation of human mesenchymal stromal cells (MSCs) with various lipid precursors, such as, polyunsaturated fatty acids, modified not only the cellular lipidome, but also the lipid composition of the EVs that were secreted.^[^ ^22^ ^]^ These observations offer potential to achieve increased control of the lipid composition present in EVs.

EVs contain relatively high levels of cholesterol. Some studies estimate the percentage to be as high as 40–60% of the total lipid content.^[^ ^7^, ^23^ ^]^ This indicates that it is highly likely that cholesterol is present in both lipid bilayers in the EVs. Furthermore, the high cholesterol content, together with the high levels of sphingolipids may imply that EVs contain lipid rafts, similar to those hypothesized for the plasma membrane. Lipid rafts are proposed regions in the membrane rich in glycosphingolipids and cholesterol, acting as a foundation for protein receptors.^[^ ^24^ ^]^ As such, they could serve an important function for the recognition between EVs and target cells, though this will require extensive experimental validation. Importantly, the outer cell membrane displays a non‐random distribution of the various lipid species present in the bilayer, also known as lipid asymmetry. This asymmetric distribution of different lipid species is well‐established with some lipids mainly found in the inner leaflet and other lipids in the outer.^[^ ^25^ ^]^ Leaflet distribution can be controlled by cellular enzymes, including flippases, floppases, and scramblases. Asymmetry also holds true for intracellular membranous structures, such as, the Golgi membrane.^[^ ^25^ ^]^ It is therefore hypothesized that EVs, both exosomes and microvesicles, may share the lipid asymmetry of their parent cell. Asymmetry in the lipid membrane of EVs could be highly beneficial for the interaction with their target cells. However, the extent to which EVs are able to maintain the asymmetry is still unclear. Analyses of Vidal et al. showed an asymmetric distribution of phospholipids in the EV membrane.^[^ ^26^ ^]^ However, no translocase activity could be found that could maintain the asymmetry. As such, it was proposed that the asymmetry is supported by the curvature of the vesicle membrane, or alternatively, the vast presence of other constituents in the membrane, such as, proteins, may interact with phospholipids to maintain the lipid distribution. In contrast, other studies reported that EVs have lost the asymmetry. As such, Laulagnier et al. found that in EVs certain lipid species have a random distribution within the lipid bilayer, for example, the phosphatidylethanolamines.^[^ ^27^ ^]^ Liposomes produced by standard methods have bilayer leaflets that are similar in composition. However, approaches are being developed that could introduce asymmetry into the liposomal lipid bilayers.^[^ ^28^ ^]^ This could aid in the development of liposomes that resemble biological membranes to a larger extent and may enhance biocompatibility.

## 3 Protein Composition of Liposomes and Extracellular Vesicles

### 3.1 Protein Incorporation into Liposomes: Proteoliposome Formation

Generally, liposomes are small artificial vesicles of spherical shape that are composed of amphiphilic lipid molecules. Due to this composition, there will classically not be any proteins present in the lipid bilayer or the intraluminal space of liposomes. However, multiple studies have been conducted on designing liposomes which incorporate proteins, so called proteoliposomes. Proteoliposomes have been extensively used as tools for biophysical studies on lipid‐protein and protein‐protein interactions, as well as, topological and topographical features of different classes of (membrane) proteins.^[^ ^29^ ^]^ In addition, approaches to engineer the pharmacokinetics and ‐dynamics of liposomes as advanced drug delivery entities are also being explored. Besides this, several bioactive ingredients derived from proteins or peptides, such as, enzymes, peptide hormones, and cytokines, have been delivered by liposomes.

Over the years, different strategies have been developed to reconstitute proteins into liposomes and these have been reviewed extensively by others.^[^ ^29^ ^]^ The various strategies differ in their efficiency and applicability for proteoliposome reconstitution. Rigaud and Lévy described several criteria that must be considered for the reconstitution of proteins, such as, the homogeneity of the protein insertion and its final orientation, the morphology, and size of the reconstituted proteoliposomes, as well as, their residual permeability.^[^ ^29b^ ^]^ There are two basic approaches for the reconstitution of proteins. The vast majority of protein reconstitution strategies are detergent‐mediated. Briefly, detergent‐solubilized purified proteins are mixed with an excess of phospholipids and appropriate detergent. Due to comicellization, lipid‐protein‐detergent, and lipid‐detergent micelles are formed. Subsequently, the amount of detergent is reduced, resulting in the formation of closed lipid bilayers in which the proteins eventually incorporate. Different approaches to remove the detergents are dialysis, gel filtration, dilution, and polystyrene beads.^[^ ^29^ ^]^ Another protein reconstitution strategy uses preformed liposomes to unidirectionally incorporate the solubilized purified proteins. In this strategy, detergent is gradually added to solubilize the preformed liposomes, followed by the addition of solubilized protein at each well‐defined step of the solubilization process. Afterward, detergent is removed from the mixture to obtain proteoliposomes.^[^ ^29^ ^]^

### 3.2 Protein Composition of Extracellular Vesicles

Whereas proteins are generally not found in liposomes, a wide range of proteins has been confirmed to be integrated in or attached to the membranes of EVs, or are present in their intraluminal space. Proteins reported to be abundant in EVs include heat shock proteins (e.g., Hsp70 and Hsp90), lysosomal‐associated membrane proteins (e.g., Lamp2a and Lamp2b), cytoskeletal proteins (e.g., actin, tubulin, and cofilin), integrins, proteoglycans, and tetraspanin proteins (e.g., CD9, CD37, CD53, CD63, and CD81).^[^ ^5^ ^]^ Furthermore, EV release and membrane trafficking from parent cells is associated with the presence of Rab GTPases (e.g., Rab4, Rab11, and Rab27) in EVs.^[^ ^30^ ^]^ In addition, EV biogenesis is reflected in the protein composition of different subsets of EVs. Biogenesis of EVs can be divided into endosomal sorting complex required for transport (ESCRT)‐dependent and ESCRT‐independent pathways. ESCRT consists of 4 protein complexes, ESCRT‐0, I, II, and III, and its accessory proteins, including for example ALG2‐interacting protein X (Alix) and tumor susceptibility gene 101.^[^ ^31^ ^]^ Therefore, these ESCRT‐related proteins are often found within EVs and used as marker proteins. On the other hand, biogenesis of EVs can originate from the ESCRT‐independent pathway. EV proteins, such as, tetraspanins, which are among the most enriched membrane proteins in EVs, play a pivotal role in ESCRT‐independent pathways.^[^ ^32^ ^]^

However, some functional proteins found in EVs may be limited to EVs isolated from specific cell sources. These cell source‐specific proteins incorporated into EVs may influence EV function. For instance, EVs derived from antigen‐presenting cells (APCs), such as, B‐lymphocytes, dendritic cells (DCs), microglia, and macrophages, are enriched with major histocompatibility complex (MHC) proteins, which are used to display foreign antigens on the surfaces of APCs.^[^ ^33^ ^]^ Research has demonstrated that EVs secreted from APCs carrying MHCs are able to stimulate CD4 positive T cells.^[^ ^34^ ^]^ Another example of cell‐specific proteins expressed in EVs was reported by Al‐Nedawi et al. Aggressive human brain tumor (glioma) cells often express a form of the epidermal growth factor receptor (EGFRvIII). In this study, glioma cell‐derived EVs expressed EGFRvIII and were responsible for intercellular transfer of EGFRvIII between glioma cells.^[^ ^35^ ^]^ In a recent study, Guerreiro and coworkers discovered, using EV proteomics, cancer‐specific protein profiles with proteins involved in processes promoting tumor progression.^[^ ^36^ ^]^ These cell‐specific proteins enriched in EVs may have a potential application as diagnostic biomarkers, or might provide more insight into the pathogenesis of several diseases.

As with lipids, the protein composition of EVs is not fully identical to that of the parent cells, suggesting the possible existence of EV protein sorting mechanisms.^[^ ^37^ ^]^ The exact mechanisms underlying specific EV protein sorting remain to be elucidated. In addition, despite the seemingly different protein profiles as a result of distinct biogenesis routes, a specific marker protein to distinguish between different EV populations still needs to be identified. A better understanding of how the protein composition of EVs is influenced by several factors, such as, biogenesis and cell source, will allow a more efficient operation of EVs as advanced drug delivery entities. In addition, a better understanding of EV protein sorting mechanisms could be used for improved control of the protein cargo that cells load into the EVs they release, and could potentially provide more control over the presence of proteins important for cellular uptake and pharmacokinetics.

## 4 The influence of Lipid Composition on Pharmacokinetics

### 4.1 Biodistribution and Clearance of Liposomes and Extracellular Vesicles

**Figure 2:**
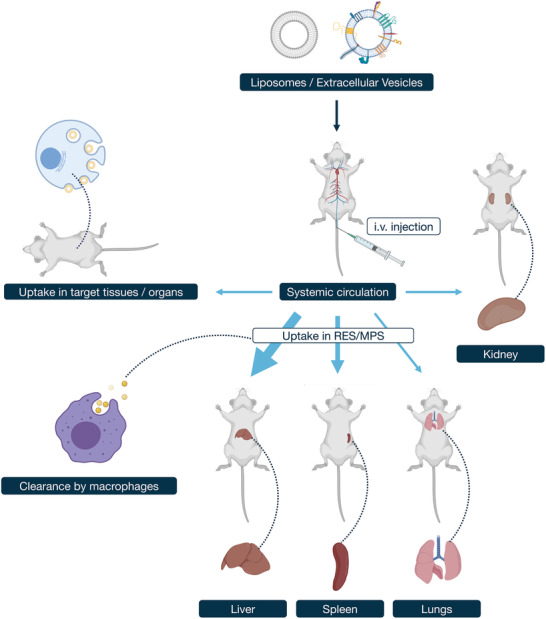
Schematic overview of pharmacokinetics of liposomes and EVs. Intravenous (i.v.) injection will deliver vesicles into the systemic circulation. Unmodified vesicles most likely are taken up by the RES/MPS, the main site being the liver, followed by the spleen. Resident macrophages in these tissues take up vesicles for clearance. Parameters can be modified by the properties of the vesicles and the administration route by which uptake can be driven to alternative target tissues (see text for further discussion).

One of the first observations made when liposomes were administered intravenously to a rodent model was their rapid clearance from the circulation.^[^ ^38^ ^]^ Subsequent tissue distribution analyses revealed that the liver was one of the main sites of uptake, followed by the spleen.^[^ ^38^ ^]^ This early sequestration may be more a consequence of physical filtration than active uptake, though ingestion by cells from the reticuloendothelial/mononuclear phagocyte systems (RES/MPS), is thought to be a key clearance mechanism. We would like to refer the reader to the upper part of Table S3, Supporting Information, for an overview of studies investigating the biodistribution of liposomes. Overall, it appears that after intravenous administration liposomes largely end up in the liver and spleen (see **Figure** **2**). It should be noted that several of the studies reported in Table S3, Supporting Information, were performed in immunocompromised rodent hosts, which may alter the immunological response toward administered vesicles and therefore does not allow immediate extrapolation to the human situation.

An overview of results from biodistribution analysis of EVs can also be found in Table S3, Supporting Information. Studies that assessed EVs, showed that intravenous injection in mice also leads primarily to a rapid accumulation in the liver, within minutes to hours, similar to that found for liposomes.^[^ ^16^, ^39^ ^]^ For the majority of studies, this appears to be independent of the cellular origin of the EVs, the purification method employed and the mouse strain that was being used. Nonetheless, some studies do support different biodistribution profiles.^[^ ^40^ ^]^ It remains to be firmly established exactly where these differences originate from, but EV composition, administration route, and dosage applied could be interesting candidates. In this respect it is worth mentioning that a large variety of labelling, imaging and analysis methods were used in the various studies. In a recent report, it was shown that this can also severely impact the biodistribution profile of EVs.^[^ ^41^ ^]^ Of note, a head‐to‐head comparison of EV biodistribution between different studies is challenging, since most studies report administered dosages as a protein concentration. Across studies, different EV isolation methods EVs were used, therefore varying in the quantity of free protein that coprecipitates with the EVs. As such, reporting protein concentrations will be less indicative for the administered dosage than particle number.^[^ ^42^ ^]^

In terms of their clearance rates, EVs appear to also have remarkable similarity to liposomes. Smyth et al. compared the clearance of EVs from MCF‐7 and PC‐3 cells with liposomes composed of phosphatidylcholine : cholesterol in a molar ratio of 67 : 33, and found that 3 h after intravenous injection, less than 5% of the administered dose remained.^[^ ^43^ ^]^ Furthermore, it was shown that EVs originating from a murine melanoma cell line were rapidly cleared from the blood circulation after intravenous injection in mice,^[^ ^39b^ ^]^ with an estimated half‐life of EVs reported to be between 2 and 4 min.^[^ ^39^, ^44^ ^]^ Similar results were recently reported by Lázaro‐Ibáñez and coworkers, who demonstrated the half‐life of EVs in the circulation of mice to be below 10 min.^[^ ^41^ ^]^ Therefore, evidence is mounting that a very limited amount of EVs is present in the circulation of mouse models after 24 h.^[^ ^39^, ^41^, ^43^, ^44^, ^45^ ^]^ The unique and complex composition of EVs in comparison with liposomes, does not seem to translate into superior circulation time. In contrast, Kamerkar et al. reported that 24 h after intraperitoneal injection EVs but not liposomes obtained from a commercial source, were detected in the circulation.^[^ ^46^ ^]^ This warrants further investigation as to whether the EV composition used in this study is distinct from others and could lead to extended circulation times. We will elaborate on this topic further in Section 5.

### 4.2 The Influence of Physicochemical Properties and Administration Route on Pharmacokinetics

#### 4.2.1 Vesicle Size

Pioneering work in the 1970s and 1980s explored how the composition and morphological characteristics of liposomes could modify the pharmacokinetic properties when applied in vivo. Juliano and Stamp compared the behavior of large MLVs with that of SUVs in their 1975 publication.^[^ ^47^ ^]^ They reported that the larger vesicles were cleared from the bloodstream approximately four times faster than the smaller vesicles in a rat model. These observations were corroborated when uptake by the rat liver was shown to be dependent on liposome size.^[^ ^48^ ^]^ Here, a comparison was made between SUVs (30–50 nm), LUVs (100–200 nm), and giant MLVs (1.3 µm), showing that larger liposomes were cleared from the circulation more rapidly than smaller sized vesicles.^[^ ^48^ ^]^ In contrast, upon local, intravitreal administration, LUVs (400 nm) showed a twofold increase in half‐life compared to SUVs (60 nm).^[^ ^49^ ^]^ Larger vesicles also seemed less effective in releasing their content in the vitreous humor of rabbit eyes.^[^ ^49^ ^]^ This would suggest that vesicle size is an important parameter to control clearance, but the optimal vesicle diameter depends on the application and accompanying administration route. In addition, cellular internalization mechanisms depend on the size of the vesicles used.^[^ ^50^ ^]^ Along with cellular uptake, the physical properties of liposomes also play a role in intracellular vesicle trafficking. Confocal microscopy imaging showed that 97.8 nm liposomes presented the highest colocalization with early endosomes, while 72.3 nm liposomes mostly colocalized with lysosomes, and are likely subjected for degradation.^[^ ^50^ ^]^ Sakai‐Kato et al. also compared liposome sizes of 101, 116, and 126 nm, but differences in uptake were not statistically significant in this study.^[^ ^51^ ^]^

For EVs, with their more polydisperse size distribution, the effects of size on pharmacokinetics are more challenging to ascertain. Zhuang et al. used a differential ultracentrifugation protocol to enrich a smaller population of exosomes and a larger population of microparticles.^[^ ^52^ ^]^ Upon intranasal administration in mice, a different biodistribution profile could be observed for these two populations. Zhang et al. used asymmetric field flow fractionation to separate different subpopulations of vesicles, including small EVs and large EVs.^[^ ^16^ ^]^ Analysis of the biodistribution of these populations showed that they varied in the accumulation in different tissues. Though these differences could be due to the differences in size, composition of these populations, such as, lipid and protein content are also likely to differ. For EVs, size and composition cannot be uncoupled as in liposomes and is therefore a challenging area to study.

#### 4.2.2 Vesicle Charge

A parameter that can be controlled in liposomes by choice of lipids is the charge on the outer surface. For unilamellar liposomes, those with a negative charge were cleared approximately four times more rapidly from the bloodstream in rats than neutral or positively charged vesicles.^[^ ^47^ ^]^ The observed slower clearance rates for positively charged liposomes may explain why these were found to associate more with tumor tissue, compared to vesicles with a near neutral and negative surface charge.^[^ ^53^ ^]^ Increased residence time could provide opportunity for vesicles to associate with other tissues. Overall, for accumulation of liposomes in tissues other than liver and spleen, clearance rate appears to be the primary factor determining whether distribution to these tissues can take place. It may also be tissue dependent which charge regime is most effective. Bajoria and Contractor studied the transfer efficiency of carboxyfluorescein encapsulated in SUVs of different charges in perfused human term placenta in vitro. Their results suggest that negatively charged liposomes are more efficient in the transfer of polar compounds in human term placenta in comparison with positively charged liposomes.^[^ ^54^ ^]^

Recently, Campbell et al. explored the use of zebrafish embryos as a model to study biodistribution of liposomes of varying charge.^[^ ^55^ ^]^ Intravenous injection of neutral, positively charged, and negatively charged formulations (≈100 nm in size) were compared. The neutral vesicles could circulate freely, whereas the negatively charged liposomes were taken up by scavenging endothelial cells and macrophages in the blood in the initial hours after injection. The positively charged vesicles remained in the circulation for a limited time, before being cleared.^[^ ^55^ ^]^ This was corroborated by Arias‐Alpizar and colleagues who developed a liposomal formulation which allows a surface charge switch of liposomes upon irradiation with UV light.^[^ ^56^ ^]^ The liposomal composition consisted of DOPC and cholesterol linked to a photoactive cholesterylamine lipid reagent in a 1:1 ratio. The liposome in its natural state presented a near neutral surface charge, allowing them to freely circulate in the zebrafish embryo and thus successfully evading the innate immune system. Upon UV irradiation, the *ζ*‐potential switched from −8 to +25 mV in less than 2 min, generating cationic liposomes. This triggered non‐specific adsorption of the vesicles to endothelial cells and their recognition by scavenging endothelial cells and macrophages. Interestingly uptake by endothelial cells and macrophages is highly dependent on the applied dose of UV light. In the intermediate charge state at low doses of irradiation, in which the surface charge shifts from near neutral to cationic, liposomes are subjected to uptake by macrophages. In contrast, at high doses uptake by endothelial cells dominates. These results not only highlight the importance of understanding how physicochemical properties of drug delivery vehicles affect pharmacokinetics, but also show potential to control them in situ.

#### 4.2.3 Lipid Formulation of the Vesicles Used

Multiple modifications to the lipid formulations of liposomes have been studied to alleviate the recognition by cells from the RES/MPS and improve circulation time to allow uptake of the vesicles into other tissues. Early work from Patel and coworkers showed that cholesterol‐content in SUVs but also in larger liposomes was an important factor determining their circulation time.^[^ ^57^ ^]^ Cholesterol enhances the stability of liposomes in the circulation, but also influences their uptake in the RES‐rich organs. It was also shown that increasing the amount of phosphatidylserine in LUVs, enhances their clearance from the blood (≈fourfold) and augments uptake in the liver (≈threefold), 2 h after injection.^[^ ^58^ ^]^ Phosphatidylserine is also one of the main phospholipids found in the EV lipidome. This phospholipid is exposed from the cell membrane during apoptosis and is known to be a trigger for uptake by macrophages. It can subsequently be recognized by specific phagocytic receptors.^[^ ^59^ ^]^ Evidence shows that this also holds true for phosphatidylserine exposed on nano‐ and microparticles. Flannagan et al. used 5 µm glass beads coated with a mixture of phospholipids to assess the importance of phosphatidylserine in the phagocytic capacity of mouse bone marrow‐derived macrophages. Presence of this lipid was crucial for uptake.^[^ ^60^ ^]^ Similar results were obtained with nanoparticles of varying compositions.^[^ ^61^ ^]^ An additional class of nanovesicles that could be relevant for drug delivery is a type made by shearing cells into small vesicles. These cell‐derived nanovesicles (CDNs) were found to have a distinctive lipid composition in comparison to cells and EVs (see Table S2, Supporting Information).^[^ ^62^ ^]^ The lower abundance of phosphatidylserine in these CDNs may reduce the clearance by macrophages, prolonging their circulation time and potentially improving cellular uptake. Whether phosphatidylserine is also relevant for uptake of EVs was examined by Matsumoto and coworkers.^[^ ^39b^ ^]^ They demonstrated that the uptake of EVs by macrophages could be reduced by using phosphatidylserine‐rich liposomes (≈100 nm in size) as a decoy. This direct competition for uptake was also seen for liposomes containing phosphatidylglycerol, but not for phosphatidylcholine‐rich liposomes. This indicates that uptake is at least partially mediated by the charge of the liposomes, which is far more negative for phosphatidylserine and phosphatidylglycerol, than for phosphatidylcholine. 4 h after intravenous injection in mice, the clearance of EVs from the blood was reduced twofold if phosphatidylserine‐ or phosphatidylglycerol‐rich liposomes were pre‐administered. Accumulation of EVs in the liver also diminished, though the overall biodistribution remained unaltered. As such, the negative charge of phosphatidylserine in the EV membrane appears to be involved in recognition and clearance of EVs by macrophages.^[^ ^39b^ ^]^ Imai et al. showed that clearance of B16BL6 EVs could be delayed when mice were pre‐treated with phosphatidylcholine:cholesterol liposomes carrying a cargo of clodronate, to deplete the macrophages.^[^ ^63^ ^]^ Also in the macrophage‐depleted animals, accumulation of EVs in the lung, liver and spleen was observed, which indicates that parenchymal cells in these organs could be taking up the EVs. Several other studies have previously reported on overcoming entrapment of liposomes in the RES/MPS using a pre‐dosing strategy. It was hypothesized that uptake by the RES/MPS could be prevented by saturating with control liposomes.^[^ ^64^ ^]^ This may subsequently improve circulation times of drug‐loaded liposomes and aid in their uptake in target tissues. However, the success of this strategy varies^[^ ^65^ ^]^ and may impair the normal function of the RES/MPS in host defense.^[^ ^5^ ^]^ For example, the stearylamine and cholesterol content in cationic liposomes leads to ROS generation in a concentration‐dependent manner in macrophage cell lines, resulting in apoptosis.^[^ ^66^ ^]^ This cytotoxic effect could be completely reversed by coating the cationic liposomes with polyethylene glycols (PEG), as will be discussed below.

Enrichment of the lipid bilayer with gangliosides and sphingomyelin was shown to reduce uptake of LUVs by macrophages^[^ ^67^ ^]^ and prolong their circulation time^[^ ^48^, ^68^ ^]^ This could also be achieved with liposomes of the LUV type mimicking the composition of red blood cell membranes, using a formulation composed of phosphatidylcholine:sphingomyelin:cholesterol:ganglioside GM1 (molar ratio 1:1:1:0.14).^[^ ^69^ ^]^ Gabizon and Papahadjopoulos compared no less than 20 different liposomal formulations and showed that the uptake by the RES/MPS differs widely between these formulations.^[^ ^70^ ^]^ This indicates that the type of lipids incorporated into the liposomal membrane play a crucial role in how the body interacts with the vesicles. In their work, the most favorable composition consisted of a small fraction of glycolipid, such as, ganglioside or phosphatidylinositol, with neutral phospholipid as the main backbone. By tweaking the liposome composition, uptake in xenografted tumors in mice could also be influenced, though the overall accumulation in tumor tissue remained well below levels in the liver.^[^ ^70^ ^]^ A very successful strategy to enhance circulation time is the decoration of liposomes with PEG.^[^ ^71^ ^]^ This topic will be discussed in more detail in a section later in this review (Section 6.1).

#### 4.2.4 Route of Administration for Vesicles

The administration route is also a parameter that can offer control over the biodistribution of both liposomes and EVs. We have listed several examples in Table S3, Supporting Information. Ivanova et al. compared the biodistribution of phosphatidylcholine:cholesterol (55:45) liposomes (≈500 nm in size) in a mouse model, after administration via intravenous (i.v.) injection or inhalation. They found that after 24 h, the liver and kidney were the main sites after i.v. injection, whereas the lungs were by far the main site of accumulation after inhalation.^[^ ^72^ ^]^ Efficient delivery of liposomes (≈100 nm in size) to the lungs was also observed by Zhao and colleagues when using inhalation as the administration route.^[^ ^73^ ^]^ Zhuang et al. examined intranasal delivery of EVs from various sources to treat inflammatory disorders in the mouse brain.^[^ ^52^ ^]^ An efficient delivery into the brain could be observed within 3 h. The authors made a distinction between exosomes and microvesicles, based on an isolation protocol using differential ultracentrifugation, and found only exosomes effective for this purpose.^[^ ^52^ ^]^ For efficient delivery to tumor tissue, Smyth et al. examined intratumoral injection of EVs derived from 4T1 murine mammary cancer cells and phosphatidylcholine:cholesterol liposomes (≈130 nm in size) with a mole percentage of 67:33. Their results show an almost exclusive distribution of both vesicle types to a 4T1 xenografted tumor. The signal observed was stronger for EVs than that of the liposomes, which may imply higher efficiency of EV retention and potentially uptake in the tumor tissue.^[^ ^43^ ^]^

### 4.3 Enhancing Vesicle Stability and Exploiting their Physical Properties

Premature release of cargo from liposomes, due to instability of the lipid bilayer has been an active area of research as it is undesirable for any DDS. Composition of the lipid bilayer is fundamental for the integrity of liposomes as well as the release kinetics of the cargo. Interaction with serum was shown to enhance liposome leakage in SUVs.^[^ ^74^ ^]^ In addition, both unilamellar and multilamellar liposomes composed of phosphatidylcholine were reported to have a one way transfer of lipid to high‐density lipoproteins in the bloodstream, which destabilizes their lipid bilayer and causes leakage of cargo.^[^ ^75^ ^]^ The addition of increasing amounts of cholesterol into the lipid bilayer of liposomes was shown to reduce leakage^[^ ^74^, ^76^ ^]^ and tailor release of encapsulated drugs.^[^ ^77^ ^]^ Cellular uptake of liposomes in target cells, may also depend on the lipid composition. Abumanhal‐Masarweh et al. reported that the internalization of liposomes into triple‐negative breast cancer cells, depends on the lipid formulation offered.^[^ ^78^ ^]^ Their results show that the characteristics of the lipid headgroup, but also of the lipid tails, are important factors in cellular uptake. Incorporation of phosphatidic acid in the lipid bilayer had the largest effect on uptake in comparison with other lipids. Potentially this is due to more specific interaction of phosphatidic acid with the surface of the target cells. Furthermore, the presence of phospholipids with short carbon tails in the liposomes, such as, DMPC and DLPC, destabilized the cell membrane of the target cells and had negative effects on cell viability. In contrast, phospholipids with longer carbon tails, such as, DPPC, supported cell proliferation, as they can integrate easily in the cell membrane. Additionally, enrichment of liposomes with cholesterol improves uptake in cancer cells, but the magnitude of the effects observed, depends also on the lipid composition.^[^ ^78^ ^]^ Bellavance and coworkers demonstrated that uptake of liposomes in glioblastoma cells was also dependent on the lipid composition.^[^ ^79^ ^]^ Non‐PEGylated cationic formulations showed the largest uptake in a 24‐h time period, whereas PEGylation was reported to inhibit cellular uptake. Cellular uptake versus lipid composition was further assessed in a study by Sakai‐Kato et al.^[^ ^51^ ^]^ Liposomes consisting of sphingomyelin:DSPC:cholesterol:DOPS at a 10:30:40:20 mole percentage were developed to mimic the lipid composition of EVs. Liposomes without sphingomyelin were also fabricated. Assessment of uptake in HeLa cells showed that sphingomyelin played only a minor role. In additional lipid compositions, DOPS was substituted for saturated DSPS. These were taken up by HeLa cells less efficiently than formulations with DOPS. DOPS was also substituted for DOPG, which shares the tail composition with DOPS, but has a different head group (see also Table S1, Supporting Information). DOPG incorporation reduced uptake by half compared to DOPS. When DOPS was omitted from the formulation altogether, uptake efficiency was also severely reduced. Therefore, in this setting, DOPS has a substantial effect on the uptake of the liposomes used and could be considered for future liposomal formulations.^[^ ^51^ ^]^

Fluidity and mechanics of the lipid bilayers are additional parameters that can be tuned in liposomes and impact the pharmacokinetics and cellular uptake. Storm et al. used liposomes, mainly MLVs, with different fluidities, one type being characterized as fluid, the other as solid.^[^ ^80^ ^]^ It was shown that the fluid liposomes were cleared from the circulation ≈80 times faster than the solid liposomes within the first 4 h. When loaded with doxorubicin (DOX), the solid liposomes turned out to have reduced efficacy as antitumor agents, as they displayed delayed antitumor activity. This was a consequence of the rate of degradation in the cell, which thus interestingly also depended on the lipid composition. As such, lysosomal degradation and subsequent release of liposome content were suggested as key factors influencing availability of encapsulated DOX.^[^ ^80^ ^]^ This was corroborated by Bakker and coworkers, who also compared fluid with solid multilamellar liposomes for delivery of ampicillin against intracellular *Listeria monocytogenes*.^[^ ^81^ ^]^ Uptake in macrophages was found to be comparable between both types of liposomes. However, with respect to degradation of the liposomes in the cells, differences were observed. The solid liposomes had a relatively slow degradation with a delayed intracellular release of ampicillin. The fluid liposomes seem more sensitive to degradation within the target cells than the solid liposomes.^[^ ^81^ ^]^ Recently, Zhao et al. varied the proportion of cholesterol incorporated in the lipid bilayer of liposomes to modify membrane fluidity. Liposomes (≈100 nm in size) with molar ratios of 80:0:5, 80:10:5, 80:20:5, and 80:40:5 of DPPC:cholesterol:DSPE‐PEG_2000_ were prepared to increase fluidity. Subsequent analysis of uptake in alveolar macrophages showed that an increase in liposomal fluidity led to a decrease in cellular uptake.^[^ ^73^ ^]^ Bompard and colleagues used the fluidity of the liposomal membrane of LUVs as a manner for selective targeting of tumor cells.^[^ ^82^ ^]^ Control of fluidity was achieved in this study by varying lipid chain length and saturation. The results reported, showed that more fluid liposomes had preferential interaction with tumor cells, whilst more rigid liposomes targeted the non‐malignant cells included in the study.^[^ ^82^ ^]^ This offers opportunity to use lipid composition itself as a targeting mechanism for drug delivery.

Another interesting physical characteristic of both liposomes and EVs that is gaining attention is their mechanical properties. The mechanics of nanoparticles were shown previously to control the extent of cellular uptake. For example, a nanoparticle system, consisting of a polymeric core, encased in a lipid shell, was reported of which the rigidity could be tuned.^[^ ^83^ ^]^ Rigidity was found to dictate the efficiency of cellular uptake. The more rigid particles were reported to traverse the cell membrane more easily than more flexible particles.^[^ ^83^ ^]^ In terms of the mechanical properties of vesicles, Liang et al. showed that incorporation of cholesterol into phosphatidylcholine liposomes decreases the deformability of the vesicles and enhances their rigidity.^[^ ^84^ ^]^ The measurements reported were obtained by atomic force microscopy measurements (AFM) to generate force curves. The Young's modulus reported increased from 1.97 to ≈13 MPa, when cholesterol was introduced, with bending moduli ranging from 0.27 × 10^19^ J to 1.81 × 10^19^ J.^[^ ^84^ ^]^ Similar findings for cholesterol incorporation were reported by Takechi‐Haraya et al. who used AFM measurements on DOTAP:cholesterol liposomes in ratios of 90:10 and 50:50.^[^ ^85^ ^]^ The bending modulus increased from 1.5 × 10^19 ^J to 2.5 × 10^19^ J with increased cholesterol. In contrast, the authors also reported analyses on liposomes composed of hydrogenated soybean phosphatidylcholine (HSPC)/PEG‐DSPE or HSPC/cholesterol/PEG‐DSPE. Here, increasing cholesterol content correlated with a decreasing in bending modulus.^[^ ^85^ ^]^ Vorselen et al. explored the effects of lamellarity on mechanics of liposomes, formulated as phosphatidylcholine:phosphatidylethanolamine:phosphatidylserine:sphingomyelin:cholesterol in a molar ratio of 15:17:8:15:45, using AFM.^[^ ^86^ ^]^ Stiffness of the vesicles was found to increase with lamellarity, with each bilayer adding 2.7 × 10^–3^ N m^−1^. Vorselen and colleagues further studied the mechanical properties of red blood cell‐derived EVs^[^ ^87^ ^]^ and 30–200 nm SUV formulations which mimic those of red blood cells^[^ ^88^ ^]^ thus allowing a direct comparison between the two vesicle types. The bending moduli of liposomes and EVs have similar values of ≈14 k_b_T and ≈15 k_b_T, respectively. The stiffness values of liposomes were 21 × 10^–3^ N m^−1^ while those of EVs ranged from 5.8 to 10.9 × 10^–3^ N m^−1^.^[^ ^87^, ^88^ ^]^ In addition, Ridolfi and coworkers characterized the mechanical properties of liposomes and EVs, using an AFM‐mode in a more high‐throughput, semiquantitative fashion using contact angle measurements.^[^ ^89^ ^]^ Liposomes of different composition varied in their deformability and therefore their stiffness. DOPC liposomes were softer than POPC, DPPC, or DSPC. Measurements on EVs from various sources showed that their deformability was comparable to the liposomes with an intermediate stiffness, indicating that from this perspective, liposomes can equal EVs. The types of EVs the authors compared, did not differ much in their mechanical properties. Similarly, Sorkin and colleagues explored the mechanical properties of EVs from red blood cells and HT1080 cell‐derived EVs, which are from fibrosarcoma origin. The authors showed, despite their cellular origin, these vesicles had comparable mechanical properties, as reflected in the binding moduli measured.^[^ ^90^ ^]^ In contrast, Zhang et al. compared the mechanical properties of small and large EVs using an AFM‐based indentation approach, and found that smaller vesicles have a higher Young's modulus (≈70–420 MPa) than larger vesicles (≈26–73 MPa) from the different cell types tested.^[^ ^16^ ^]^ Therefore, mechanical properties of vesicles likely depend on size and composition, and may be easier to tune for liposomes than for EVs.^[^ ^89^ ^]^ The mechanical properties of EVs and liposomes were also studied by Sakai‐Kato et al. using AFM. The stiffness of EVs derived from HepG2 cells, was reported to be 26 × 10^–3^ N m^−1^, whilst that of the studied liposomes ranged from 16 to 32 × 10^–3 ^N m^−1^.^[^ ^51^ ^]^Liposomes incorporating the DOPS lipid were slightly softer than those with DSPS. The authors also showed that there may be a connection between mechanics and uptake.^[^ ^51^ ^]^ The DOPS‐containing liposomes incidentally showed enhanced uptake in HeLa cells compared to the DSPS containing liposomes.^[^ ^51^ ^]^ For EVs, mechanics could be important for cellular interaction as well. Whitehead et al. compared the nanomechanical properties of non‐malignant HCV‐29, malignant non‐metastatic T24 and malignant metastatic FL3 bladder cell‐derived EVs and their implications in endothelial disruption.^[^ ^91^ ^]^ Although their sizes were similar, the malignant cell line‐derived EVs had reported values of 95 and 280 MPa, while values for the non‐malignant EVs were a higher at 1527 MPa. Surprisingly, although both metastatic and non‐metastatic EVs were able to disrupt an endothelial monolayer, the latter, with reduced stiffness, had a greater effect. Additionally, EVs from malignant cell types showed lower adhesion, which aided in their travel across the endothelial monolayer more readily. On the other hand, the non‐malignant EVs did not have any effect in endothelial disruption.^[^ ^91^ ^]^ Choosing appropriate vesicle mechanics for drug delivery is therefore also a parameter that should be included. With respect to the mechanical properties of vesicles, it is important to emphasize that performing and analyzing these measurements is not trivial. Though the various studies reported in this section all use AFM as the assessment method, the parameters analyzed differ greatly, reporting Young's moduli, bending moduli and stiffness measurements. As such, comparisons between the different studies are challenging to make. Furthermore, differences in methodology, such as the experimental approach and the model used to fit the data obtained, are likely to cause considerable inconsistencies in values between the different studies reported. As vesicle mechanics is an area of research that is only beginning to be explored in depth, the methodologies applied will likely need optimization to generate accurate measurements. We would like to refer to recent reviews for topical insights into methodology for mechanical characterization of vesicles.^[^ ^88^, ^92^ ^]^

## 5 Functional Pharmacokinetic‐Related Proteins in Native Extracellular Vesicles

**Figure 3:**
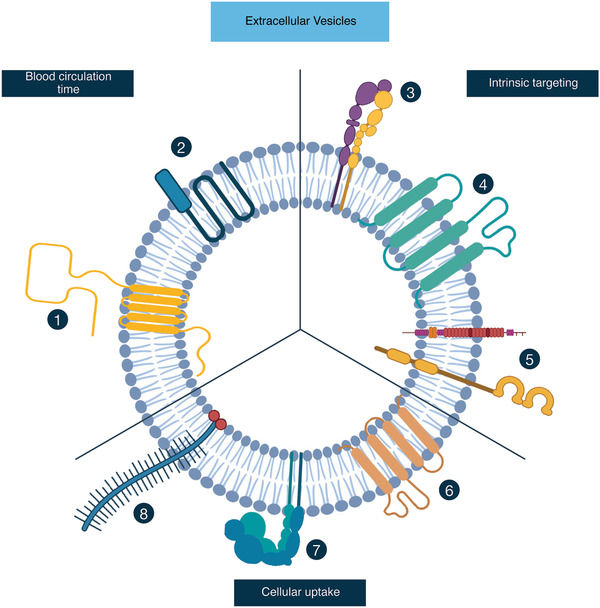
Naturally occurring EV‐surface proteins that alter circulation time, intrinsic targeting, and cellular uptake. The presence of 1) CD47 or 2) CD55/CD59 on the membrane of EVs can prolong the blood circulation time by evading phagocytic clearance. EVs show intrinsic targeting capacity that is associated with the incorporation of 3) integrins, 4) tetraspanins, and 5) other proteins, such as, fibronectin and Wnt4, on the surface of EVs. The 6) tetraspanin, 7) integrin, and 8) proteoglycan compositions of EVs affect their uptake into recipient cells.

For therapeutic purposes, favorable pharmacokinetics of EVs are essential to deliver therapeutic cargo to diseased tissue in the body. In contrast to their artificial counterparts, an advantage of using EVs as advanced drug delivery entities is that they already contain several proteins that may contribute to their pharmacokinetic behavior in vivo. The importance of the presence of proteins on the surface of EVs on pharmacokinetics and biodistribution profile, was demonstrated by Charoenviriyakul et al.^[^ ^93^ ^]^ Upon intravenous administration, EVs treated with proteinase K exhibited a significantly smaller volume of distribution, as compared to untreated EVs.^[^ ^93^ ^]^ Since proteinase treatment digests proteins located on the surface of EVs, these results suggest the involvement of surface proteins in pharmacokinetics in terms of reducing volume of distribution and their biodistribution. In this section we will describe various proteins that are natively present in or on EVs, and contribute to their pharmacokinetics. **Figure** **3** shows a schematic overview of various pharmacokinetic‐related proteins that have been found to be present in EVs.

### 5.1 Blood Circulation Time and Protein Composition

EVs are praised for their capability to prolong the blood circulation time of their (therapeutic) cargo in vivo, often attributed to the “immunologically privileged” status of EVs. However, much remains unexplored about the in vivo properties of EVs, including blood circulation time, tissue distribution, and clearance dynamics. These parameters largely determine therapeutic effectiveness and potential toxicity in a clinical setting.

The hypothesis of EVs escaping phagocytic clearance, and thereby evading the immune system, would contribute to their status of advantageous advanced DDS with prolonged circulation time. However, as has been observed with their artificial counterparts, administered EVs are rapidly cleared from the blood circulation by macrophages in the liver and spleen (see Section 4). Nonetheless, there is evidence that some EVs express surface proteins that prevent their uptake by macrophages, such as CD47, a “do not eat me” signal. CD47 is a putative marker of “self” that gives macrophages a “do not eat me”‐signal, by which macrophage discriminates between “foreign” and “self” cells. Phagocytes bind to CD47 with signal regulatory protein *α*, resulting in a dephosphorylation cascade to suppress the phagocytic activity of macrophages.^[^ ^94^ ^]^ CD47 has been found on the surface of EVs secreted by fibroblasts, T cells, and MSCs.^[^ ^46^, ^95^ ^]^ Kamerkar et al. demonstrated that 3 h after intraperitoneal injection, EVs from CD47‐knockout mice showed ≈twofold reduced retention in vivo, whilst EVs with high levels of CD47 expression displayed higher retention in the circulation.^[^ ^46^ ^]^ These results suggest a superior escape from phagocytic clearance of EVs, while the extent to which EVs evade clearance requires further research. Recently, Belhadj and coworkers reported a study in which they developed a combined CD47‐based “eat me”/’do not eat me’‐strategy in an effort to evade rapid clearance by the RES/MPS. Briefly, cationized mannan‐modified DC‐derived EVs (M‐EVs) were administered to satiate the RES/MPS (“eat me”‐strategy). Subsequently, nanocarriers loaded with antitumor drugs and a homing peptide were fused to CD47‐enriched EVs (“do not eat me”‐strategy) and administered via the tail vein of mice. The hybrid nanocarriers demonstrated prolonged circulation time and increased tumor accumulation in vivo, attributed to the saturation effect of M‐EVs on liver and spleen accumulation, and the RES/MPS escape effect of CD47‐enriched EVs in the hybrid nanocarriers.^[^ ^96^ ^]^ In addition to CD47 as a possible contender for evading the immune system, EVs secreted by APCs and retinal pigmented epithelium have been found to express CD55 and CD59.^[^ ^97^ ^]^ Membrane‐bound molecules, such as CD55 and CD59, are capable of protecting cells from homologous complement lysis by inhibiting the complement pathways at several points.^[^ ^98^ ^]^ Clayton et al. demonstrated that antibody blocking of CD55 and CD59 on the surface of EVs resulted in significantly increased lysis of EVs, indicating that EVs escape complement‐mediated lysis through the expression of CD55 and CD59, which contributes to their stability and prolonged blood circulation time.^[^ ^97a^ ^]^ However, it is not clear to what extent these proteins contribute to evading the clearance of EVs, and thus prolonging the circulation time of allogenic administered EVs in a clinical setting. Moreover, in order to be an auspicious therapeutic delivery system, EVs must demonstrate pharmacokinetic properties superior to more readily available artificial liposomes and other lipid‐based nanoparticles.

### 5.2 Intrinsic Targeting Capacity of Native Extracellular Vesicle Proteins

In addition to having a sufficient blood circulation time, efficient targeting of EVs toward specific target cells contributes to exerting its therapeutic function and the transfer of functional cargo. EVs are often described as having intrinsic targeting properties upon in vivo administration, at least to some extent, as lipid and protein composition of EVs can influence tropism toward specific organs or disease sites. Below, we will describe several native proteins that have been associated with the intrinsic targeting capacity of EVs.

Integrins are well‐conserved cell adhesion receptors for extracellular matrix (ECM) proteins that support cell adhesion and drive cell migration. Operating integrin molecules are heterodimers compiled of two integral membrane glycoprotein subunits.^[^ ^99^ ^]^ The intrinsic targeting capacity of EVs has in some cases been linked to the presence of integrins on the surface of EVs, as has been supported by several studies from the cancer field. Hoshino et al. demonstrated that EVs derived from distinct cancer cell lines, that have the propensity to metastasize to the lung (MDA‐MB‐231 and 4175) or to the liver (BxPC‐3 and HPAF‐II), primarily accumulated to the preferential metastatic organs of their cells of origin, after in vivo administration. In addition, proteomic analysis of distinct EVs revealed distinct integrin expression patterns, in which the integrins *α*6*β*4 and *α*6*β*1 were associated with lung metastasis and *α*v*β*5 related to liver metastasis. EVs isolated from integrin *β*4 knockdown 4175 cells and integrin *β*5 knockdown BxPC‐3 cells showed reduced accumulation in the lungs and liver, respectively.^[^ ^100^ ^]^ These findings were further supported by Charoenviriyakul and coworkers, who demonstrated that B16BL6 melanoma cell‐derived EVs express integrin *α*6*β*1, an integrin known to be involved in lung targeting. After proteinase K treatment, lung distribution of EVs was significantly reduced after intravenous injection. Integrin *α*6*β*1 was degraded after proteinase K treatment, suggesting that integrins play a key role in the intrinsic targeting capacity of EVs.^[^ ^93^ ^]^ Another example of tissue‐specific targeting of EVs was demonstrated by Park and colleagues. The presence of integrin *α*4*β*7 at the surface of gut‐tropic T cell‐derived EVs was shown to determine their distribution to the villi of the small intestine under psychological conditions.^[^ ^101^ ^]^

In addition to integrins, tetraspanins have also been associated with intrinsic targeting capacities of EVs. After intravenous administration, EVs containing Tspan8 accumulated in the pancreas of mice, whereas coexpression of integrin *β*4 led to an enrichment in the lungs.^[^ ^102^ ^]^ In addition, findings by Yue et al. demonstrated the involvement of EV tetraspanins CD151 and Tspan8 in the formation of metastases in different tumor systems. Knockdown of CD151 and Tspan8 eliminated the metastatic capacity of rat pancreatic adenocarcinoma tumor cells, whereas pre‐treatment with EVs derived from highly metastatic wild‐type pancreatic cells regained the metastatic potential.^[^ ^103^ ^]^ These results suggest that the presence of CD151 and Tspan8 on tumor EVs is essential for targeting sites for the formation of novel metastases. Furthermore, in a recent study, Laulagnier and colleagues demonstrated that a specific subset of EVs derived from neuro‐2a cells, which lack tetraspanin CD63, specifically bind to dendrites of neurons, unlike EVs carrying CD63, which bind to both neurons and glial cells.^[^ ^104^ ^]^ Although no in vivo studies on targeting were performed, these results support the concept of the involvement of tetraspanins and the intrinsic targeting capacity of EVs.

Another example of an EV surface protein that has been implicated in the intrinsic targeting of EVs is fibronectin. Fibronectin was abundantly present on the surface of microvascular endothelial cell‐derived EVs, and was considered to be involved in targeting the EVs toward oligodendrocyte precursor cells via an interaction with heparin sulfate proteoglycans.^[^ ^105^ ^]^ Banfai and colleagues associated Wnt4 on EVs derived from thymic epithelial cells with the in vivo accumulation of EVs in the thymus.^[^ ^106^ ^]^ Tropism for the thymus was further enhanced by overexpression of Wnt4 in the parent cells, whereas control Wnt5‐transgenic EVs were outperformed in their targeting capacity.^[^ ^106^ ^]^ Another example was demonstrated by Wu et al. who showed that EVs released by lung metastatic hepatocellular carcinoma exhibit lung tropism with diverse distributions to other important organs in immunocompetent mice.^[^ ^107^ ^]^ Proteomic analyses revealed SLCO2A1 and CLIC1 expression on non‐tropic EVs conferred lung tropism, whereas CD13 induced spleen tropism. In addition, redirected tropisms significantly diminished EV accumulation in multiple other organs, including the liver, kidneys, heart, and brain.^[^ ^107^ ^]^

Altogether, these studies suggest, to some extent, that there is evidence for cell‐ and organ‐specific tropism of EVs from various sources. However, whether EVs are equipped with proteins to selectively interact with specific cell types or tissues is still debated. Most studies suggest that the degree of innate tropism is very limited, and indicate that the organs of RES/MPS, such as, the liver and spleen, are the predominant sites of EV accumulation upon exogenous administration (see Section 4). Hence, as has been studied extensively with artificial liposomes, EVs may need to be manipulated, for drug delivery purposes, to improve target specificity of EVs and reduce their uptake by other cells.

### 5.3 Cellular Uptake and Native Extracellular Vesicle Proteins

For EVs to act as advanced DDS of therapeutic nucleic acids and proteins, it is essential that EVs are internalized by the recipient cell and deliver their cargo into the cytoplasm in order to elicit a therapeutic effect. There is overwhelming evidence, both direct and indirect, that EVs are internalized by cells and deliver their cargo into recipient cells. Despite the rapidly growing interest in exploiting EVs as DDS, the exact effectors responsible for the functional transfer of EV‐cargo remain to be elucidated. One of the effectors that might be responsible, at least to some extent, for the internalization of EVs are proteins present on the surface of EVs, as was demonstrated by Escrevente et al.^[^ ^108^ ^]^ After proteinase K treatment, uptake of EVs derived from SKOV3 cells, an ovarian carcinoma cell line, was inhibited by 45%, indicating that the proteins are required for the uptake of EVs.^[^ ^108^ ^]^ Here, we will review several examples of proteins that have been shown to participate in the internalization of EVs and cytoplasmic delivery of their cargo.

Before, we mentioned tetraspanins being involved in the EV biogenesis and tropism. However, accumulating evidence suggests that tetraspanins might also play a direct or indirect role in EV attachment and uptake into recipient cells. One of the first works supporting this comes from the reproduction field, where, in a loss‐of‐function study, CD9^–/–^ female mice were shown to be infertile. In CD9^–/–^ female mice, sperm‐oocytes binding was standard, yet sperm‐egg fusion was almost entirely prevented in CD9^–/–^ oocytes.^[^ ^109^ ^]^ In a follow‐up study, infertility was partly restored by exogenously administered mouse and human CD9, suggesting the involvement of CD9 in the fusion of sperm with oocytes.^[^ ^110^ ^]^ Furthermore, exogenously administered CD9‐containing EVs derived from oocytes have also been shown to contribute to the fusion of sperm and oocytes from CD9^–/–^ mice.^[^ ^111^ ^]^ In addition to the reproductive field, CD9 has shown to be entangled in the facilitation of EV uptake by numerous human cell lines, including HEK293, HeLa, SH‐SY5Y, and B and T lymphocytes. Böker and coworkers demonstrated that ineffective EV‐encapsulated lentiviral transduction could be circumvented by overexpression of CD9 in the absence of any pseudotyping viral glycoprotein or fusogenic molecule.^[^ ^112^ ^]^ The involvement of tetraspanins in the fusion of EVs with the recipient cells is mostly elucidated by the use of specific antibodies, leading to a steric block that prevents the interaction between both entities. For instance, Morelli et al. demonstrated that blocking the tetraspanins CD9 and CD81 on the surface of bone marrow‐DCs, using antibodies, resulted in a decrease in EV uptake.^[^ ^113^ ^]^ Furthermore, in an antibody blocking study, blockade of CD9 and CD81 on EVs derived from several cell sources interfered with binding to the recipient cells.^[^ ^114^ ^]^ In addition to antibody blocking studies, Rappa et al. demonstrated that knockdown of CD9 in two different cancer cell lines abrogated the uptake of the secreted EVs by recipient cells. Reciprocally, the cellular uptake of CD9‐positive EVs by CD9‐knockdown recipient cells was significantly diminished.^[^ ^115^ ^]^ Interestingly, addition of CD9 monoclonal antibodies to recipient cells during their incubation with EVs resulted in a significantly enhanced internalization of EVs. These findings were supported by Santos and colleagues, who demonstrated that the presence of CD9 antibodies resulted in an increase of cytoplasmic uptake of CD9‐GFP‐EVs by recipient cells. In contrast, the same group reported that addition of Fab fragments of CD9 antibodies to recipient cells during incubation with EVs interfered with EV internalization.^[^ ^116^ ^]^ The authors attributed the increased uptake in the presence of CD9 antibodies to CD9 cross‐linking, which facilitates the binding of CD9‐overexpressing EVs to the membrane of recipient cells, whereas CD9 Fab fragments interfered with these cellular processes.

In addition to CD9, other tetraspanin‐complexes have been reported to be involved in internalization of EVs by recipient cells. For instance, Tspan8 is known to form complexes with integrins, such as the reported complex Tspan8‐CD49d, which contributes to EV uptake into rat aortic endothelial cells. Antibody blocking of CD151 and CD49c prevented EV binding to the recipient cells, whereas antagonistic antibody treatments indicate that CD106 strengthens the interaction between EVs and the recipient cell.^[^ ^117^ ^]^ Furthermore, Zhao and colleagues demonstrated that EVs derived from knockout CD151 or Tspan8 mice resulted in impaired uptake of EVs by endothelial cells and methylcholanthrene‐induced tumor cells.^[^ ^118^ ^]^ These findings were supported by Yue et al., who reported that knockdown of CD151 and Tspan8 in pancreatic adenocarcinoma cell line ASML resulted in the secretion of EVs that showed a severely impaired uptake by leukocytes.^[^ ^103^ ^]^ These data suggest a role for tetraspanins in the uptake of EVs by recipient cells.

In tetraspanin enriched microdomains (TEMs), in addition to tetraspanins being abundantly present, other adhesion molecules and transmembrane receptor proteins are located in raft‐like structures in the plasma membrane of EVs. Within these TEMs, tetraspanins are often found to form complexes with integrins, which have also been shown to play a role in EV uptake.^[^ ^119^ ^]^ For example, Altei and coworkers studied the role of *α*v*β*3 integrin in the uptake of MDA‐MB‐231‐derived EVs by breast epithelial cells. In this study, EV uptake was significantly diminished after the EVs were incubated with DisBa‐01, a disintegrin inhibitor with specificity for *α*v*β*3 integrin.^[^ ^120^ ^]^ In accordance with these findings, Al‐Dossary et al. demonstrated that EVs derived from murine oviductal fluid express *α*v*β*3 integrin and uptake of these EVs into mouse sperm was significantly inhibited after incubation with either RGD‐peptides—a promiscuous integrin binding site—or anti‐*α*v antibodies.^[^ ^121^ ^]^ In another study, knockdown or blocking of exosomal ITG*β*4 reduced EV uptake significantly in the lung, whereas knockdown of ITG*β*5 and pre‐incubation with RGD‐peptide or anti‐ITG*α*v*β*5 antibody significantly diminished EV uptake by the liver.^[^ ^100^ ^]^ These data suggest that, next to EV tropism, integrins expressed on the surface of EVs are involved in the uptake of EVs by recipient cells.

In addition, in a reciprocal fashion, expression of integrins on the surface of the recipient cells also plays a pivotal role in the uptake of EVs. Antibody blockade of the binding sites of CD11a or its ligand ICAM‐1 on the surface of DCs diminishes the uptake of EVs. Furthermore, blocking the integrins *α*v and *β*3 on the surface of DCs obtained similar results.^[^ ^113^ ^]^ In another study, Fuentes and coworkers demonstrated that silencing the integrin ITGB3 using shITGB3 in recipient cells resulted in decreased EV uptake, whereas the origin of the vesicles—either from shCON of shITGB3 cells—did not affect their uptake into recipient cells.^[^ ^122^ ^]^ Zech et al. demonstrated that tumor‐derived EVs were preferentially incorporated into CD11b+, CD11c+, CD44+, CD49d+, and CD54+ leukocytes, whereas EV uptake was significantly diminished after leukocyte pre‐incubation with antibodies against CD11b, CD11c, CD44, CD49d, and CD54.^[^ ^114^ ^]^ Furthermore, the uptake of hepatic stellate cell (HSC)‐derived EVs was blocked by pre‐treatment of HSCs with RGD peptide, integrin *α*v or *β*1 siRNAs, or integrin *α*v*β*3 or *α*5*β*1 neutralizing antibodies, indicating that integrins mediate the binding of EVs to HSCs.^[^ ^123^ ^]^ In line with these findings, Dabbah et al. recently demonstrated that uptake of MSC‐derived EVs into multiple myeloma cells was dependent on integrins *α*3 and *β*1, as uptake of EVs was significantly reduced after incubation with RGD peptide, natalizumab, and anti‐CD29 monoclonal antibody.^[^ ^124^ ^]^ Another study showed that integrin *β*3 is involved in binding and uptake of EVs by Ly6C^high^ monocytes in the lung after incubation with *β*3 integrin receptor blocking peptide.^[^ ^125^ ^]^

Besides tetraspanins and integrins, other proteins, such as, proteoglycans, have been associated with EV uptake. Proteoglycans are a heterogeneous family of macromolecules that consist of a core protein and one or more covalently attached polysaccharide‐chains.^[^ ^126^ ^]^ For instance, CD44, a hyaluronic acid binding glycoprotein, was found to be enriched on the surface of EVs derived from cancer cells and, subsequent to EV uptake, EVs induced a change in cellular morphology of human peritoneal mesothelial cells. When CD44 expression was knocked down in the parent cancer cells, the uptake and observed effects induced by EVs was significantly reduced.^[^ ^127^ ^]^ In addition, GFP‐HAS3‐overexpressing MV3 cell‐derived EVs showed a significantly diminished uptake into recipient cells after hyaluronan oligosaccharides treatment. Since hyaluronan oligosaccharides compete with hyaluronan binding, these data suggest that EV binding and uptake is partly regulated by CD44.^[^ ^128^ ^]^ Next to CD44, heparan sulphate proteoglycans (HSPGs), which are associated with the uptake of viral particles and lipoproteins, have shown to be expressed on the surface of EVs.^[^ ^129^ ^]^ Bladder cancer cell‐derived EVs were internalized by human bladder cancer cells, whereas uptake was partially blocked by heparin treatment.^[^ ^130^ ^]^ Interestingly, treatment of EVs with heparinase to remove surface proteoglycans did not affect the uptake of EVs.^[^ ^129^ ^]^ These results suggest that HSPGs on the recipient cell surface are essential for the uptake of EVs, whereas EV‐HSPGs might not be as important.

Taken together, there is a tremendous amount of evidence available that suggests that EVs express different proteins on their membrane that are involved in the adhesion of EVs to the surface of the recipient cells. Knocking down or sterically blocking proteins on the membrane surface of EVs or recipient cells, such as, integrins and tetraspanins, results in a diminished uptake of EVs. A specific area that would benefit from further investigation is EV uptake, since there is currently no consensus on the main mechanism of EV internalization. Furthermore, the fate of EV‐cargo after EV uptake into the recipient cell is usually not assessed, which is probably why no conclusions have yet been drawn regarding the relationship between EV‐mediated cargo transfer and functional consequences. Nonetheless, EV entry into cells through internalization followed by entrapment into the lysosomal lumen, as was observed by their artificial lipid counterparts such as, liposomes, is found to be the most putative mechanism.^[^ ^131^ ^]^ In this pre‐published paper, EVs encapsulated with CD63‐*β*‐lactamase were efficiently internalized by recipient cells, whereas *β*‐lactamase substrate cleavage was not reported. In contrast, EVs modified to carry a viral fusogenic protein (VSV‐G), showed functional transfer of *β*‐lactamase, suggesting native EVs exhibit low endosomal escape efficiency.^[^ ^131^ ^]^ Thus, EV‐cargo is presumably subjected to a sequential acidic environment, leading to restricted cytoplasmic delivery due to degradation of EV‐cargo. Next to the facilitation of adhesion of EVs to recipient cells, it appears to be unlikely that adhesion proteins, such as, tetraspanins and integrins, play a direct role in EV‐cargo transfer and endosomal escape. It appears to be more likely that other molecules on the surface of EVs are involved with the latter mechanism, hence further research is needed to establish whether these proteins or other molecules on the surface of EVs are responsible. Of note, most studies exploring the uptake of EVs into recipient cells, whereas endosomal escape and functional intracellular cargo release is usually not assessed. It is of great importance to study the mechanisms in which EVs are internalized as this may be pivotal for functional outcomes of EVs administered as advanced DDS. Besides, native proteins in EVs could serve as inspiration for the decoration of liposomes to tweak pharmacokinetics (see Section 6.2).

## 6 Engineering Liposomes and Extracellular Vesicles to Tweak Pharmacokinetics

### 6.1 Polyethylene Glycol: A Toolset to Prolong Circulation Time

For liposomes and EVs to be advantageous DDS, it is of great importance to deliver adequate concentrations to target tissues. However, the formation of a protein corona surrounding nanoparticles in the circulation, followed by opsonization and induced clearance from blood by the RES/MPS, results in a substantial diminution in bioavailability. Prolonging the blood circulation time of liposomes and EVs allows for extensive contact time of the vesicles with targeted cells, and therefore largely affects the therapeutic potential of liposomes and EVs. One of the most widely studied approaches to enhance blood circulation time of nanoparticles is by coating the surface with PEG. PEG is an inert hydrophilic polymer and shields nanoparticles from aggregation, opsonization, and cellular uptake by macrophages, thereby prolonging the systemic blood circulation time.^[^ ^132^ ^]^

Decorating the surface of liposomes with PEG has been extensively studied, and resulted in the first FDA approval of a PEGylated nanoparticles product, exceptionally improving the circulation half‐life.^[^ ^133^ ^]^ One of the first works supporting prolongation of circulation time due to the incorporation of PEG into large unilamellar liposomes was reported by Klibanov et al., who demonstrated a significant increase in the blood circulation half‐life (*t* = 5 h) as compared to non‐PEGylated large unilamellar liposomes (*t* < 30 min). In addition, PEGylation of liposomes did not impact their integrity.^[^ ^71^ ^]^ Similarly, Zhang and colleagues reported that PEGylated liposomes encapsulated with salvianolic acid B exhibited prolonged circulation time in the blood, as compared to free salvianolic acid B and non‐PEGylated liposomes.^[^ ^134^ ^]^ Effective surface shielding of liposomes was demonstrated to be proportional to PEG polymer chain length. PEGylated liposomes decorated with 750 Da PEG‐chains revealed comparable blood circulation time with non‐PEGylated liposomes, whereas liposomes PEGylated with 5 kDa chains significantly enhanced circulation time.^[^ ^135^ ^]^ In contrast, Dos Santos and coworkers reported prolonged circulation time, irrespective of the incorporated PEG‐chain length.^[^ ^136^ ^]^ The variation in to what extent different PEG‐chain lengths affect blood circulation time is probably closely related to physicochemical properties of liposomes and their surface charge. PEGylation with both 750 Da and 5 kDa PEG‐chains inhibited the clearance of positively charged stearylamine liposomes, whereas only decorating negatively charged phosphatidic acid‐liposomes with 5 kDa PEG‐chains inhibited clearance. Interestingly, functionalization with none of the PEGs inhibited the clearance of negatively charged phosphatidylserine‐liposomes. These results lead to the hypothesis that different sizes of opsonins mediated the clearance of specific liposomal formulations, which can be circumvented by shielding the nanoparticles using PEG, keeping in mind the liposome formulation and PEG‐chain length.^[^ ^137^ ^]^ In addition to PEG‐chain length, PEGylation density has been associated with aggregation potential of liposomes, revealing higher densities (10 mol%) is much less prone to aggregation, as compared to lower PEGylation densities (5 and 3 mol%).^[^ ^138^ ^]^

Based on the rationale that PEG shields liposomes from interactions with plasma proteins and improves circulation time, researchers have decorated the surface of EVs with PEG‐molecules. For instance, Kooijmans and coworkers coated EVs derived from Neuro2a cells or platelets with targeting ligands conjugated to PEG to improve EV characteristics for drug delivery to tumor cells. In their work, upon intravenous administration, EVs engineered with PEG were detectable in plasma for longer than 60 min post‐injection, whereas unmodified EVs were rapidly cleared from the circulation within 10 min.^[^ ^45b^ ^]^ Similar results were reported in a study from Shi et al., who designed copper‐64 (^64^Cu)‐radiolabeled PEG‐decorated EVs. Without the shielding capacities of PEG, ^64^Cu‐EVs exhibited very short blood circulation time, whereas PEGylated ^64^Cu‐EVs revealed prolonged blood circulation and reduced hepatic clearance.^[^ ^139^ ^]^ Another study investigated the targeting abilities from paclitaxel (PTX)‐loaded EVs engineered with aminoethylanisamide‐PEG vector moieties to target the overexpressed sigma receptor in lung cancer cells. Targeted EVs exhibited superior therapeutic outcomes which were partly attributed by the authors to improved circulation time in the blood, allowing the EVs to target pulmonary metastases.^[^ ^140^ ^]^ Similarly, Antes and colleagues cloaked the EV surface with a DMPE phospholipid membrane anchor conjugated to PEG and streptavidin to alter EV biodistribution in vivo. Although blood circulation time was not assessed, intravenously administered PEGylated revealed altered biodistribution patterns.^[^ ^141^ ^]^

Despite the fact that PEGylation of liposomes and EVs seems to prolong blood circulation time, PEGylated nanoparticles exhibit some drawbacks as well, such as the hindrance in cellular uptake and endosomal escape by the attached PEG‐chains.^[^ ^132^ ^]^ Furthermore, anti‐PEG antibody production has been reported upon repeated injections of PEGylated liposomes. The formation of anti‐PEG antibodies augments clearance, and is referred to as the accelerated blood clearance (ABC) effect.^[^ ^142^ ^]^ Although the ABC‐effect has not been investigated in relation to PEGylated EVs, it is plausible that this effect also applies to PEGylated EVs.

### 6.2 Engineering Pharmacokinetics of Liposomes with Proteins

Owing to a myriad of favorable characteristics, liposomes have attracted substantial attention as DDS, especially after the first FDA‐approved liposome based therapeutic: Doxil. However, as described before, (uncoated) liposomes have non‐specific adsorption with various opsonins, such as plasma proteins and cells which form a protein corona that surround the particles when circulating in vivo. Due to the opsonization of these nanoparticles, they are easy to be degraded, easily scavenged by macrophages, and have low targeting specificity.^[^ ^143^ ^]^ Nowadays, in order to obtain more safe and effective liposomes in vivo, research aims to incorporate function‐specific membrane proteins or peptides into liposomal membranes. These recombinant proteoliposomes appear to be a promising strategy to provide liposomes with increased circulation, active targeting capacity, and augmented cellular uptake. Below, we discuss several examples of the incorporation of function‐specific proteins to alter the pharmacokinetic characteristics of liposomes, whilst engineering of EVs is discussed in Section 6.3.

#### 6.2.1 Prolonging Blood Circulation Time of Liposomes

Pre‐coating liposomes with dysopsonic proteins, to avoid the formation of a protein corona surrounding the nanoparticles in the circulation, is a widely studied approach to prolong blood circulation time. For instance, Yokoe et al. evaluated the in vivo disposition characteristics of coupled recombinant human serum albumin (HSA) onto the surface of PEGylated liposomal DOX in sarcoma‐bearing rats. Compared to PEG‐liposomal DOX and free DOX, the bioavailability of HSA‐PEGylated liposomal DOX was significantly higher, as indicated by the area under the curve (AUC), being 89.7 µg h^−1^ mL^−1^. The AUC of PEGylated liposomal DOX and free DOX were 33.8 and 4.52 µg h^−1^ mL^−1^, respectively. Furthermore, the tissue clearance was significantly lower for the HSA‐PEGylated liposomal (7 mL h^−1^), compared to the clearance of the PEGylated liposomal DOX and free DOX, being 17.9 and 131 mL h^−1^, respectively.^[^ ^144^ ^]^ Another example of prolonged circulation time in vivo was reported by Furumoto et al., who coated the surface of PEG‐modified liposomes with rat serum albumin (RSA). After intravenous administration, RSA‐PEGylated liposomes showed significantly longer blood‐circulating properties and smaller hepatic clearance, compared to PEGylated liposomes.^[^ ^145^ ^]^ Interestingly, Peng et al. demonstrated, by measuring the particle size, that there was no influence on the anti‐adhesion properties of phospholipid‐nanoparticles and bovine serum albumin (BSA)‐phospholipid‐nanoparticles after 2 h incubation with BSA as well as diluted mouse serum.^[^ ^146^ ^]^ In addition, Vuarchey et al. used BSA to coat PEGylated small unilamellar liposomes as an approach for macrophage specific drug delivery. In vivo, splenic macrophages internalized BSA‐coated liposomes more quickly as compared to conventional liposomes and PEGylated liposomes.^[^ ^147^ ^]^ These results suggest that coating liposomes with albumin would augment the clearance from the blood circulation. Another reported strategy is to coat DOX‐loaded liposomes with thermally denatured BSA, as demonstrated by Jung and colleagues. Compared to BSA‐coated liposomes, a reduction in opsonin protein binding to denatured BSA‐coated DOX‐loaded liposomes in rat blood plasma, measured by particle size, was observed, indicating an augmented stability in plasma.^[^ ^148^ ^]^ Furumoto et al. proposed several theories on how albumin reduces the opsonization of PEGylated liposomes: 1) By blocking the binding sites for serum proteins on the liposomal surface; 2) by enhancing the hydrophilicity of the liposomal surface; 3) by altering the structure of PEG molecules into a “brush” conformation.^[^ ^145^ ^]^ In line with the above‐mentioned research, Giulimondi et al. proposed another strategy of pre‐coating unilamellar liposomes with an artificial corona made of human plasma proteins. In vitro incubation of these engineered liposomes with whole blood reduces elimination by leukocytes, and shows great promise as a strategy to prolong circulation in vivo.^[^ ^149^ ^]^

Interestingly, it is not clear whether a protein corona is being formed on the surface of EVs. Recently, Palviainen et al. compared the EV proteome to that of nanoparticles which had been incubated with plasma to generate a protein corona. This comparison revealed that, in total, 89 of the “EV corona proteins” were corresponding to those belonging to the corona on nanoparticles. This similarity suggests that certain plasma proteins are tightly incorporated onto the surface of EVs and form a protein corona.^[^ ^150^ ^]^ Furthermore, melittin and CM15, two membrane active peptides with antimicrobial effect, drastically affect the protein corona adsorbed to red blood cell‐derived EVs, further suggesting the presence of a protein corona.^[^ ^151^ ^]^ Further research investigating the in vivo emergence of a protein corona on the surface of (exogenously) administered EVs and the consequences in regard to EV clearance, is required.

By applying beneficial features of EVs and other naturally occurring nanoparticles to liposomes, enhanced pharmacokinetic properties can be achieved. For instance, CD47, a glycoprotein belonging to the immunoglobulin superfamily, is expressed on mammalian cell membranes and EVs. Kamerkar et al. reported that increased retention of EVs in the blood circulation of mice is likely due to CD47‐mediated protection of EVs.^[^ ^46^ ^]^ The ability to evade clearance by macrophages makes CD47 a suitable option to engineer the surface of nanoparticles to enhance blood circulation time. One of the first works supporting this comes from the cancer field, where Rodriquez et al. demonstrated that CD47 and the functional fragment of CD47 conjugated on synthetic beads reduced phagocytic uptake and prolonged circulation time of the nanobeads, as compared to control beads and PEGylated beads.^[^ ^152^ ^]^ This theory was further confirmed in liposomes by Tang et al. by coating the liposomal surface with a CD47‐derived peptide ligand. After administration, the “self”‐peptide engineered liposomes adsorbed onto hepatic phagocytes, shielding the subsequently injected poly(lactic‐*co*‐glycolic acid) nanoparticles from the RES/MPS. Compared with conventional liposomes, the elimination half‐life of nanoparticles subsequent to administered “self”‐peptide liposomes was significantly increased, resulting in an improved residence time in circulation.^[^ ^153^ ^]^ Moreover, Hayat et al. reported a significant prolonged circulation time of DOX‐loaded liposomes coated with a CD47 mimicry peptide after intravenous injection in mice, as compared to PEGylated DOX‐containing liposomes. Furthermore, unwanted accumulation of DOX in the liver, spleen, kidney, and heart were significantly diminished by engineering the liposomal surface with the CD47 mimicry peptide.^[^ ^154^ ^]^ Decorating the surface of nanoparticles with CD47 reduced cellular internalization across M0, M1, and M2 macrophage populations. Interestingly, a pronounced reduced uptake of CD47 coated nanoparticles was observed in M1 macrophages, compared to M0 or M2 macrophages, suggesting that nanoparticle evasion of specific components of the immune system may be enabled with CD47.^[^ ^155^ ^]^

#### 6.2.2 Active Targeting of Liposomes

In general, active targeting aims to increase the bioavailability at the site of diseased tissue and to minimize off‐target accumulation and effects. Many of the studies dedicated to investigate the application of active targeting liposomes are aimed at cancer treatment, with fewer studies applying active targeted liposomes for tissue repair and other diseases. Compared with healthy cells, tumor cells display upregulated tumor‐specific receptors, such as, human epidermal receptor‐2 (HER2). The most widely studied approach of active liposomal targeting is by the conjugation of the liposomal surface with targeting moieties, such as receptor‐specific ligands and monoclonal antibodies (immunoliposomes). Below, we discuss several examples of active targeted liposomes.

Overexpression of HER2 plays a significant role in the progression in a variety of solid tumors, whereas overexpression in breast cancer is most renowned.^[^ ^156^ ^]^ Eloy and colleagues coated PTX and rapamycin (RAP) coloaded liposomes with an anti‐HER2 monoclonal antibody, and evaluated the therapeutic efficacy in HER2‐positive tumor‐bearing mice, as compared to control solution, free PTX/RAP solution, and PTX/RAP coloaded liposomes. Anti‐HER‐2‐coated immunoliposomes were superior in controlling tumor growth in vivo, with tumor volume averages in accordance with 25.27%, 44.38%, and 47.78% of tumor volume of untreated control, free PTX/RAP solution and PTX/RAP coloaded liposomes, respectively. In vitro experiments demonstrated enhanced cytotoxicity induced by anti‐HER2‐coated immunoliposomes, which was attributed to augmented cellular uptake mediated by HER2‐binding.^[^ ^157^ ^]^ In another study, anti‐HER2‐coated PEGylated immunoliposomes were compared with unmodified liposomes for delivery of PTX to HER2‐positive breast cancer in a mouse model. Immunoliposomes showed superior tumor tissue distribution of PTX and enhanced antitumor efficacy in vivo.^[^ ^158^ ^]^ In addition, Rodallec et al. demonstrated in vivo tumor accumulation ranging from 3% up to 15% of the total administered dose of anti‐HER2‐coated docetaxel‐liposomes in MDA‐MB‐453 and MDA‐MB‐231 tumor‐bearing mice. Moreover, when compared to free docetaxel + free trastuzumab, tumor growth was reduced by 89% (MDA‐MB‐453) and 25% (MDA‐MB‐231).^[^ ^159^ ^]^

In addition to active targeting of liposomes toward HER2, other tumor‐specific targets are being explored. For instance, Chi et al. used IL‐4R‐binding peptide‐1 (IL4RPep‐1) labeled DOX‐loaded liposomes to target the overexpressed interleukin‐4 receptor in H226 tumor‐bearing mice.^[^ ^160^ ^]^ Compared to unlabeled DOX‐loaded liposomes, the IL4RPep‐1‐labeled liposomes accumulated significantly more in the tumor and had greater anti‐tumor activity.^[^ ^160^ ^]^ Gholizadeh and colleagues reported prolonged blood circulation and significant increased half‐lives in tumor tissue of sepantronium bromide (YM155) loaded into PEGylated large unilamellar liposomes with or without coating of the liposomal surface with SATA‐modified monoclonal anti‐disialoganglioside (GD2) antibodies, as compared to free YM155.^[^ ^161^ ^]^ Interestingly, a clear added therapeutic value of using anti‐GD2 immunoliposomes instead of PEGylated liposomes could not be demonstrated in the conducted study.^[^ ^161^ ^]^ Another example of the formulation of chemotherapeutic drugs into targeted liposomes was reported by Wang et al.^[^ ^162^ ^]^ In this study, CD59 antibody‐conjugated miRNA‐1284/cisplatin‐loaded liposomes (CD/LP‐miCDDP) were designed for augmented therapeutic efficacy in cervical cancers. Compared with non‐targeted liposomes, CD/LP‐miCDDP showed a significantly higher cytotoxicity in HeLa cells in vitro, but no inferior pharmacokinetic properties, with regard to AUC and clearance, were demonstrated in vivo.^[^ ^162^ ^]^ Finally, Shein et al. used vascular endothelial growth factor (VEGF) and its receptor type II (VEGFR2) as a target for active targeting of cisdiamminedinatratoplatinum‐loaded liposomes toward glioma cells.^[^ ^163^ ^]^ Antibodies against VEGF and VEGFR2 were conjugated to the liposomal surface, and revealed prolonged blood circulation in glioma C6‐bearing rats, as compared with free drug. However, compared with non‐targeted PEGylated liposomes, targeted liposomes displayed substantial decrease in blood concentration and augmented clearance in vivo. Shein et al. attributed this effect by enhanced removal by the RES/MPS as a result of the decorated liposomal surface with antibodies.^[^ ^163^ ^]^

Whether the active targeting of liposomes by coating the surface with monoclonal antibodies and other receptor‐specific peptides will meaningfully impact the doses that can be used or reduce systemic side‐effects is still debated. It is important to highlight that a systemic evaluation of the biodistribution of active targeting liposomes is required. Currently, it remains unclear whether the accumulation of immunoliposomes at the desired area is guaranteed in comparison to other non‐targeted organs. Although several studies have demonstrated effective improved targeting of liposomes to a tumor or target organ, other studies still report a high non‐specific accumulation in the classical clearance organs, leading to loss of effective dose available upon in vivo administration. For instance, antibody‐directed targeting against HER2 did not increase tumor accumulation of small unilamellar immunoliposomes, as both targeted and non‐targeted liposomes achieved similarly high concentrations in HER2‐overexpressing breast cancer xenografts, being 7–8% injected dose per gram tumor tissue.^[^ ^164^ ^]^ Another example where no difference in tumor uptake was found between anti‐HER2 targeted immunoliposomes and non‐targeted liposomes, being 1.9% and 1.7% at the end of treatment, respectively, was reported by Rodallec and coworkers.^[^ ^165^ ^]^ Roveri et al. used a rhabdomyosarcoma (RMS)‐specific peptide to actively target vincristine‐loaded liposomes in a RMS xenograft mouse model. Targeted liposomes were slightly more enriched in the RMS‐tumors, compared to control liposomes. However, it was demonstrated that the major organs of liposome biodistribution remained the spleen and the liver.^[^ ^166^ ^]^ Moreover, Sugiyama et al. demonstrated that the liposome biodistribution of several single‐ and dual‐targeted liposomes was highest in the kidneys, spleen, and liver, and not in the tumor of the tumor‐bearing mice.^[^ ^167^ ^]^ Interestingly, integrin *β*6‐targeted immunoliposomes loaded with 5‐fluorouracil (5‐FU) showed enhanced tumor targeting properties in vivo, as compared with non‐targeted liposomes and free 5‐FU. However, the 5‐FU concentration remained still high in the liver, which was attributed to accelerated drug depletion as a result of liver metabolism.^[^ ^168^ ^]^

Taken together, active targeting of liposomes still yields variable results with regard to enhanced therapeutic anti‐tumor activity in vivo. Furthermore, in some cases, active targeting of liposomes did not exhibit more specific tumor distribution, suggesting that passive targeting of liposomes might be the main relevant mechanism. It is likely that the enhanced therapeutic efficacy observed in active targeted liposomes are at least partially explained by differences in liposome‐tumor cell interactions. Following this rationale, it has been suggested that immunoliposomes achieved augmented intracellular drug delivery via monoclonal antibody‐mediated endocytosis and thus enhanced therapeutic efficacy. To facilitate the translation of the targeted liposomes as superior drug delivery entities, this is an area requiring more research and in‐depth evaluation.

#### 6.2.3 Augmenting Cytoplasmic Delivery Efficiency of Liposomes

Upon reaching the tumor cells or diseased tissue, the therapeutic cargo loaded inside liposomes has to be delivered and released into the cytoplasm or nucleus in order to evoke a therapeutic effect. In general, several mechanisms have been associated with the uptake of liposomes, such as, clathrin‐dependent endocytosis, caveolin‐dependent endocytosis, and micropinocytosis.^[^ ^169^ ^]^ Overall, liposomes frequently become trapped in the lysosomal lumen. Here, the main part of their cargo is degraded by a sequential acidic environment, leading to limited efficiency of cytoplasmic delivery. In order to efficiently internalize liposomal cargo into the target cells, liposomes have been engineered to escape the endocytosis‐lysosomal pathway.

A biological entity that is well‐known for its ability to efficiently internalize cargo into the cytoplasm of target cells are viruses. It has been reported that the lipid envelope of viruses is equipped with fusion peptides, such as hemagglutinin, that are responsible for fusion with the endosomal/lysosomal membrane. After fusion, the virus releases its cargo into the cytoplasm and thereby escapes endosomal degradation.^[^ ^170^ ^]^ Inspired by this endosomal escape mechanism, GALA peptide was designed to mimic the function of hemagglutinin to promote acidic destabilization of the endosomal membrane. GALA is a pH‐dependent fusogenic peptide composed of 30 amino acids and has been successfully incorporated into liposomes.^[^ ^171^ ^]^ For instance, Santiwarangkool et al. demonstrated that liposomes modified with GALA‐peptide are internalized into human lung endothelial cells via a clathrin‐mediated pathway. In this study, the percentage of lung endothelial cells and alveolar type 1 epithelial cells with internalized DiD‐labelled GALA‐liposomes was significantly higher, as compared to PEGylated liposomes. Moreover, in vivo podoplanin knockdown activity of GALA‐liposomes encapsulating podoplanin siRNA is significantly enhanced, as compared to untreated mice, further suggesting that enhanced cytoplasmic delivery is accomplished by GALA‐liposomes.^[^ ^172^ ^]^ In addition, Akita and colleagues reported significant differences in cytoplasmic delivery of cytokine signaling 1 (SOCS1) siRNA delivered by multifunctional envelope‐type nano‐devices (MEND) modified with GALA. In bone marrow‐derived dendritic cells, SOCS1‐siRNA loaded GALA‐MEND significantly suppressed endogenous gene expression and successful endosomal escape was established, as compared to unmodified MEND.^[^ ^173^ ^]^ In another study Sakurai et al. developed a shorter version of GALA (shGALA) and combined PEGylated MEND with shGALA for systemic siRNA delivery to tumors. siRNA encapsulated in shGALA‐MEND resulted in 82% knockdown of target gene and endosomal escapes were enhanced, as compared to unmodified MEND. Furthermore, in vivo administration showed a significant inhibitory effect on tumor growth, demonstrating that engineered liposomes with GALA have great potential for more efficient cytoplasmic delivery.^[^ ^174^ ^]^

In addition to using hemagglutinin inspired GALA to enhance cytoplasmic delivery of liposomal encapsulated cargo, other fusogenic peptides are currently being explored for enhanced endosomal escape. For instance, Tu et al. designed a synthetic analogue of glycoprotein H, a fusogenic peptide from herpes simplex virus, and incorporated it into the surface of lipofectamine‐pGL3 encapsulated liposomes. In vitro evaluation showed a significantly enhanced cellular uptake and up to 30‐fold increase in the transgene expression in human cell line.^[^ ^175^ ^]^ Alternatively, Weecharangsan et al. demonstrated a significantly more efficient cellular uptake of HSA‐coated liposomes loaded with antisense oligodeoxyribonucleotide G3139, as compared to uncoated liposomes.^[^ ^176^ ^]^ In line with this observation, Chen et al. designed dual‐targeting lipid nanoparticles by coating the surface with folic acid‐modified BSA, and reported both in vitro significant augmented cytotoxicity and cellular uptake induced by the encapsulated coumarin in the vesicles.^[^ ^177^ ^]^ The enhanced cellular uptake induced by albumin was proposed to be the result of albumin protonation upon acidification of the endosomes. Subsequently, the protonated albumin is able to interact with the endosomal membrane and induce its fusion with the liposomes or induce destabilization resulting in enhanced cytoplasmic delivery.^[^ ^176^ ^]^

Despite the sanguine achievements of these strategies to enhance intracellular delivery, endosomal escape offers limited control in the efficiency by the heterogeneity of the endosomal environment.^[^ ^178^ ^]^ In light of these limitations, recent research has focused on an alternative delivery route that uses the direct access to the cellular cytoplasm provided by gap junctions. Gap junctions are used to directly transfer small molecules, such as ions, second messengers, and metabolites in cytosol from the donor cell to a neighboring cell.^[^ ^179^ ^]^ In addition, Soares and colleagues showed that connexin 43 (Cx43), an important gap junction protein, is present in EVs obtained from various sources in the form of hexameric channels, and are responsible for the interaction and transfer of cargo between EVs and recipient cells.^[^ ^180^ ^]^ Connexins are a critical component of cellular gap junctions, and have been incorporated into liposomes. Kaneda et al. designed proteoliposomes containing Cx43 using cell‐free translation systems with plasmids encoding Cx43. In this study encapsulated calcein and NEMO‐binding domain peptide was efficiently transferred into Cx43 expressing cultured cells. This transfer was blocked in the presence of a gap junction inhibitor and in Cx32‐expressing cells.^[^ ^181^ ^]^ Furthermore, in another study evaluating the exploitation of tight junctions to enhance cellular uptake efficiency, AT1002, a peptide that opens tight junctions, was used to efficiently deliver siRNA‐encapsulated small unilamellar liposomes, both in vivo and in vitro.^[^ ^182^ ^]^ Of note, efforts to enhance cytosolic delivery of mRNA mediated by EVs engineered with a constitutively active Cx43 S368A mutant are currently being explored with promising results.^[^ ^183^ ^]^

### 6.3 Protein Engineering Extracellular Vesicles to Enhance Pharmacokinetics

As described earlier, there is a vast body of evidence supporting that EVs possess many advantageous pharmacokinetic features compared to other artificial lipid‐based DDS. In part, these favorable characteristics are ascribed to the functional proteins present on the surface of EVs. However, in addition to functional proteins being innately present within EVs, approaches to engineer and improve the pharmacokinetic properties of EVs are also being explored. In general, there are two strategies by which moieties can be added to the surface of EVs. Proteins expressed on the surface of EVs can be modified through genetic and metabolic engineering of the secreting cells, for instance by the transfection of EV producing cells with vectors encoding for an EV transmembrane protein fused to a protein or peptide. Alternatively, the surface of EVs has been engineered post‐isolation through chemical engineering, such as click chemistry, which has been shown to apply proteins and peptides in a controllable manner.^[^ ^184^ ^]^ In this section, we will review several examples of approaches where EVs have been modified with functional proteins to alter the pharmacokinetic properties of EVs as an advanced DDS.

#### 6.3.1 Prolonging Circulation Time of Extracellular Vesicles

Prolonging the blood circulation time of EVs will enable the EVs to travel to the target tissues and increase the probability of efficient cargo delivery into the recipient cell. As stated before, exogenously administered EVs exhibit a rather short half‐life, with the majority of EVs being cleared within 60 min after injection.^[^ ^185^ ^]^ To the best of our knowledge, few studies have investigated the incorporation of functional proteins to modify the blood circulation time of EVs. However, Kamerkar and colleagues reported that through overexpression of CD47 on the surface of EVs, blood circulation time was increased. After intraperitoneal administration, EVs with high levels of CD47 showed higher retention in the blood circulation, as compared to CD47 knockout EVs or unmodified EVs.^[^ ^46^ ^]^

#### 6.3.2 Active Targeting of Extracellular Vesicles

Several studies have shown that EVs exhibit intrinsic tropism toward specific organs or diseased sites depending on proteins expressed on the surface of EVs. Although the degree of innate tropism is very limited, altering the surface of EVs with targeting moieties could potentially impact biodistribution and targeting capabilities of EVs. Following this rationale, several studies have looked into active targeting of EVs through modifying the surface to express different proteins, including antibodies, ligands, and peptides (see Table S4, Supporting Information).

To review the difference between active targeting of EVs and liposomes, we will elaborate on several examples that used the same peptide to alter the in vivo fate of both biological and artificial vesicles. Ohno et al. engineered HEK293 cells to express the EGFR‐targeting GE11‐peptide fused with the transmembrane domain of the platelet‐derived growth factor receptor. EVs derived from these modified cells were loaded with the tumor suppressor gene let‐7a and systemically administered into epidermal growth factor‐expressing breast cancer mice.^[^ ^186^ ^]^ Upon administration, GE11‐engineered EVs showed, compared with unmodified EVs, a pronounced increase in tumor accumulation and exhibited an antitumor effect mediated by let‐7a.^[^ ^186^ ^]^ Although accumulation of EVs in other organs was not quantified, the depicted results showed a substantial accumulation of both targeted and untargeted EVs in the liver. Similarly, Cheng and colleagues investigated the in vivo targeting capacities of DOX‐loaded liposomes expressing the GE11‐peptide.^[^ ^187^ ^]^ Briefly, lipids of soy phosphatidylcholine and cholesterol at a molar ratio of 2:1 were utilized to prepare liposomes using the thin film hydration method, followed by incubation with DOX. Subsequently, DSPE‐PEG_2000_‐GE11 or DSPE‐PEG_2000_ were incorporated into the DOX‐loaded liposomes following the post insertion method. An EGFR‐overexpressing tumor xenograft model in mice was used to study the in vivo targeting capacities of the liposomes. Upon intravenous administration, both types of liposomes were mainly concentrated in the liver and spleen, but gradually decreased with increasing circulation time. Compared with the unmodified liposomes, active targeting of liposomes resulted in increased accumulation and prolonged retention in tumor tissue. Although the in vivo tumor‐killing activity was not examined, it was demonstrated that liposomes containing the GE11‐peptide showed the highest cytotoxic activity in vitro.^[^ ^187^ ^]^ Both studies indicate that active targeting of vesicles results in increased tumor accumulation in vivo. Moreover, these studies suggest that liposomes and EVs exhibit similar biodistribution behavior, regardless of active targeting moieties expressed on the surface, with the highest accumulation found in the liver. However, this does not mean that the biodistribution behavior of both types of vesicles is interchangeable with each other. For instance, Wiklander and coworkers reported that DC‐derived EVs engineered with a rabies virus glycoprotein (RVG)‐targeting moiety expressed on the surface accumulated mostly in the liver, spleen, and GI‐tract, and significantly increased brain and heart accumulation compared with unmodified EVs.^[^ ^39c^ ^]^ On the other hand, liposomes carrying the RVG‐peptide showed that the vast majority accumulated in the liver and kidneys.^[^ ^188^ ^]^ Although biodistribution behavior differs between liposomes and EVs, several reports show that functionalization of the surface of both liposomes and EVs with a targeting moiety, such as, an *α*v integrin‐specific RGD peptide, results in increased tumor accumulation, but does not seem to circumvent accumulation in clearance organs.^[^ ^189^ ^]^ Of note, it is challenging to perform a head‐to‐head comparison between liposomes and EVs, due to differences in lipid‐composition, EV heterogeneity, imaging techniques, and animal models, among others.

Altogether, engineering of EVs with targeting moieties has demonstrated promising results in improving the EV delivery to a specific organ or diseased site. However, the core rationale behind active targeting is to ensure delivery of EVs and their cargo to the sites of therapeutic action whilst circumventing accumulation at off‐target sites. As depicted in Table S4, Supporting Information, modifying the vesicle surface with targeting proteins or peptides does not seem to evade off‐target accumulation in clearance organs, such as the liver, spleen, or kidneys. Upon in vivo administration, non‐specific accumulation in these clearance organs will presumably lead to a dramatic loss of efficacious dose. Besides, it should be considered that such targeting strategies may compromise the function and structure of EVs, or potentially trigger immune responses and reduce biocompatibility.

#### 6.3.3 Augmenting Cytoplasmic Delivery Efficiency of Extracellular Vesicles

For EVs to be effective advanced DDS, they must productively interact with recipient cells in target tissue leading to functional transfer of EV‐cargo. For instance, functional transfer of EV‐cargo has been reported by Al‐Nedawi et al., who showed that EGFRvIII can be “shared” between glioma cells by intercellular transfer through EVs.^[^ ^35^ ^]^ There is growing compelling evidence that suggests that, upon EV internalization, EVs release their cargo or are destined for lysosomal degradation, depending on the presence of molecules on the surface of EVs. As such, EVs are expected to be internalized by their recipient cells via similar mechanisms as their artificial, liposomal counterparts.^[^ ^190^ ^]^ Besides enhancing targeting characteristics, EVs have been engineered to express peptides to promote the internalization of EVs into cells. Below, we will review several approaches that have been examined to augment cytoplasmic delivery by engineering functional proteins on the surface of EVs.

Peptides have been used to decorate the surface of EVs to induce uptake of EVs by specific pathways. For example, Nakase et al. modified HeLa‐derived EVs with a stearylated K4‐peptide with high affinity for the model receptor E3‐EGFR expressed on the surface of recipient HeLa cells. The K4‐engineered EVs induced E3‐EGFR‐receptor clustering and promoted internalization via macropinocytosis. In addition, loading the modified EVs with ribosome‐inactivating protein saporin resulted in enhanced cytotoxicity in MDA‐MB‐231 (E3‐EGFR) cells in vitro, compared with unmodified EVs.^[^ ^190^ ^]^ In another study, Nakase et al. modified EVs with an arginine‐rich cell‐penetrating peptide (CPP) to induce active macropinocytosis for efficacious EV uptake. Engineered EVs activated macropinocytosis pathways, and increasing the number of arginine residues in the CPP‐peptide enhanced the cellular EV uptake efficiency. Interestingly, engineered EVs showed improved delivery of the encapsulated ribosome‐inactivating protein saporin and effectively attained anti‐cancer activity.^[^ ^191^ ^]^

Another strategy uses anti‐HER2 single chain variable fragment (scFv) domains to decorate the surface of EVs and increase the uptake of the engineered EVs into HER2‐positive cancer cells. Longatti and coworkers demonstrated that high‐affinity anti‐HER2‐scFv on EVs and cells overexpressing HER2 showed the highest EV uptake.^[^ ^192^ ^]^ In line with this data, Kooijmans et al. demonstrated that decoration of EVs with anti‐EGFR nanobodies increased the uptake by EGFR‐overexpressing tumor cells, whereas uptake was not affected in EGFR‐negative Neuro2A cells.^[^ ^193^ ^]^ In addition, EVs displaying GE11 showed increased uptake by MCF‐7 cells, whereas neuropeptide Y display did not affect the uptake. In contrast, neurotensin and urokinase plasminogen activator on the surface of EVs diminished uptake by MDA‐MB‐231 cells.^[^ ^194^ ^]^ Interestingly, anti‐HER2 modified EVs colocalized with markers known to be taken up by macropinocytosis, caveolin‐mediated and clathrin‐mediated endocytosis, whereas wild‐type EVs and irrelevant scFV‐engineered EVs colocalized with markers representative for caveolae‐mediated endocytosis and macropinocytosis, respectively.^[^ ^192^ ^]^ These data suggest that the intracellular trafficking of EVs can be modulated by the proteins expressed on their surface, which might increase effective cargo transfer by circumventing endosomal degradation.

As we have seen before in the engineering of liposomes to augment cytoplasmic delivery of cargo, science draws its inspiration from biologic entities, such as, viruses. For instance, Temchura and colleagues demonstrated that incorporation of the G protein of vesicular stomatitis virus (VSV‐G) into EVs significantly increased their uptake, improved cargo transfer, and induced maturation of DCs.^[^ ^195^ ^]^ In addition, Yang et al. showed that incorporating VSV‐G into the surface of EVs promoted efficient transfer of GFP‐tagged CD63 or glucose transporter 4 (GLUT4) to plasma membranes, both in vitro and in vivo. Furthermore, GLUT4 transferred by EVs increased glucose uptake of recipient cells, suggesting the potential of incorporating viral peptides to augment cytoplasmic delivery by EVs.^[^ ^196^ ^]^ In addition to VSV‐G, GALA‐peptide, a negatively charged pH‐sensitive fusogenic peptide, has been engineered on the surface of EVs, as was also performed in liposomes (see Section 6.2.3.). Nakase and coworkers incorporated GALA‐peptide and cationic lipids that act as a “glue” to improve cellular uptake of EVs and efficient cytosolic release of their cargo. Saporin encapsulated in engineered EVs showed significant cytotoxic activity against cancer cells, whereas this effect was almost absent when unmodified EVs were applied. Interestingly, in the absence of cationic lipids, cellular uptake efficiency of GALA‐EVs was significantly diminished, causing low efficacy of cargo release within the recipient cells.^[^ ^197^ ^]^ Compellingly, cationic lipids were not required to significantly improve the internalization of PEGylated GALA‐liposomes with encapsulated podoplanin siRNA.^[^ ^172^ ^]^ Moreover, it is believed that PEGylation of EVs and liposomes reduces the uptake by recipient cells, probably due to the PEG chains blocking interaction of surface proteins. Although a head‐to‐head comparison between these studies is perilous, these data suggest that GALA‐peptide influences the internalization and efficient transfer cargo of liposomes more profoundly, as compared to EVs.

Taken together, improving efficacious delivery of EV‐cargo into living cells in vivo is of the utmost importance for EVs to be advantageous DDS, especially when the cargo contains biological molecules, such as, proteins, DNA, or RNA. Despite the continued growing body of evidence and methods, the introduction of engineered proteins for improved EV internalization can still be limited by endosomal entrapment of EV‐cargo. For instance, decorating the surface of EVs with (parts of) antibodies might increase the uptake of EVs, the fate of EV‐cargo inside the cell is, however, still uncertain since this process most likely does not bypass the potential of becoming entrapped in an endosome. However, functional transfer of EV‐cargo has been demonstrated, which suggests that at least some subpopulations of EVs could be trafficked to lysosomes for degradation, while other subpopulations may merge with endosomal membranes and deliver their cargo. Further research is needed to establish which molecules on the surface of EVs determine what happens to EVs after they are internalized by the cell. In contrast, the development of fusogenic EVs carrying peptides such as VSV‐G or GALA‐peptide shows tremendous potential, since endosomal entrapment is mostly circumvented. However, the introduction of viral proteins might introduce other difficulties, such as, immunogenic reactions to administered EVs.

### 6.4 Altering Pharmacokinetics Using Protein‐Related Strategies

**Figure 4:**
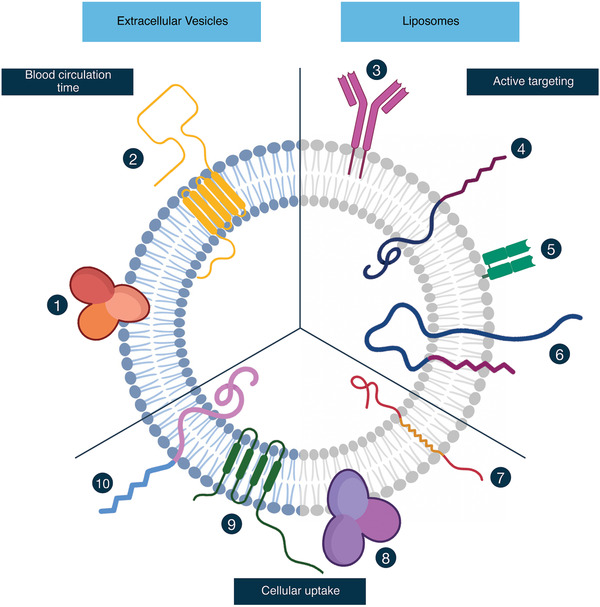
Overview of proteins used to tweak pharmacokinetics of liposomes and EVs. The blood circulation time of liposomes and EVs can be prolonged by the incorporation of 1) dysopsonic proteins, such as, albumin, and 2) phagocytic clearance evading proteins, such as, CD47. Liposomes and EVs have been actively targeted toward tumors and diseased tissues by the incorporation of 3) antibodies, 4) targeting peptides, 5) antibody‐fragments, and 6) targeting proteins. 7) Cellular uptake and subsequent cytoplasmic delivery of therapeutic cargo has been enhanced by decorating the surface of liposomes and EVs with virus‐inspired peptides, such as, GALA‐peptide. Moreover, cellular delivery has been enhanced by incorporating 8) albumin, 9) gap‐junction proteins such as, CxC43, and 10) other fusogenic peptides.

Altogether, different strategies to tweak pharmacokinetics in regard to circulation time, targeting specific disease sites, and cellular uptake, using protein‐related strategies have been widely studied, as depicted in **Figure** **4**. The pharmacokinetic behaviors of both liposomes and EVs have been shown to be tunable, showing great promise for future applications as advanced DDS. Despite these advances, many aspects remain to be elucidated in order to translate these findings into clinically successful therapeutics. For example, decorating the surface of both liposomes and EVs with CD47 has shown to prolong blood circulation time. However, the impact of these alterations on the surface of these nanoparticles in relation to dosage, administration frequency, and clinical readouts, remains to be fully elucidated. Furthermore, in the case of active targeting strategies of liposomes and EVs, numerous approaches fail to circumvent high non‐specific accumulation in the classical clearance organs, such as, the liver. The rationale behind active targeting of vesicles is to reduce whole‐body dosage, diminish side‐effects, and enhance on‐target efficacy. Whether these requirements are currently achieved in a clinical setting is unclear. Of note, off‐target accumulation of liposomes and EVs in the liver could possibly function as a depot for sustained release, which could potentially highly affect the whole‐body dosage and administration frequency. In addition, it is still unclear what determines the fate of therapeutic cargo after uptake of liposomes and EVs by the recipient cells. Upon internalization, therapeutic cargo could potentially be degraded in lysosomes or productively delivered into the cytoplasm of the recipient cells and induce alterations in the phenotype of target cells. In order to utilize liposomes and EVs more effectively in a therapeutic context, it would be highly beneficial to gain more insight into the underlying biological mechanisms, and how these mechanisms could be exploited. At this moment, various aspects of blood circulation time, active targeting, and cellular uptake of liposomes and EVs have been elucidated, and a tremendous amount of research has focused on methods by which these features may be altered to produce advantageous lipid‐based DDS. Since there is a lot of overlap between in vivo behavior and engineering potential of liposomes and EVs, there are a lot of lessons that can be learned from both fields.

## 7 Non‐Protein Related Active Targeting Strategies

**Figure 5:**
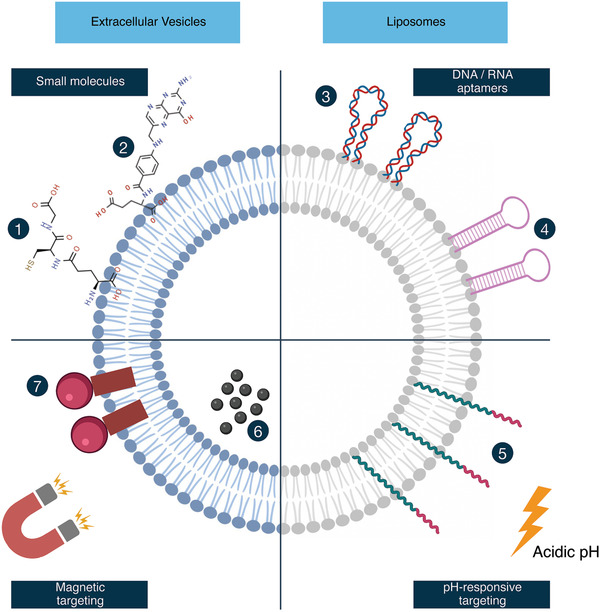
Overview of non‐protein related strategies to actively target liposomes and EVs. The surface of liposomes and EVs has been decorated with various small molecules, such as, 1) glutathione and 2) folic acid, to actively target diseased tissue. In addition, targeting of vesicles has been demonstrated by incorporating 3) DNA and 4) RNA aptamers on the vesicle surface. 5) Moreover, the acidic environment of tumor tissue has been exploited to target liposomes and EVs using pH responsive polymers. 6,7) Finally, liposomes and EVs have been modified with iron‐based nanoparticles to obtain magnetic‐responsive vesicles, which can be targeted by applying an external magnetic field.

For a long time, research has focused on actively targeting EVs and liposomes toward specific tissues. Surfaces of both vesicles have been decorated with a wide array of antibodies, antibody fragments, peptides, and other protein‐related targeting moieties. However, other strategies, including small molecules, nucleic aptamers, magnetic targeting, pH responsive targeting, and sugar moieties, among others, have been employed to ensure delivery of EVs and liposomes to their sites of therapeutic action while avoiding accumulation at off‐target sites. A schematic overview of various non‐protein related targeting strategies is depicted in **Figure** **5**.

### 7.1 Small Molecules as Targeting Moieties

Small molecules have been incorporated into EVs and liposomes to enhance accumulation at the target site. The folate receptor is overexpressed on the surface of the vast majority of cancer tissues, whereas its expression is limited in healthy tissue.^[^ ^198^ ^]^ Following this rationale, Manugala et al. formulated bovine milk‐derived EVs carrying the tumor‐targeting ligand folic acid (FA) and loaded withaferin A (WFA) into the luminal space. Compared to vehicle (PBS), unmodified EVs, and WFA‐EVs, FA‐targeted WFA‐EVs demonstrated, upon oral gavage, significantly higher growth inhibition in mice bearing human lung cancer xenografts.^[^ ^199^ ^]^ Unfortunately, biodistribution studies revealing the fate of administered FA targeted WFA‐EVs in vivo were not performed. In a similar manner, Soe and coworkers synthesized FA‐conjugated liposomes encapsulating both celastrol and irinotecan for targeted breast cancer therapy. Upon intravenous administration, FA‐conjugated liposomes showed superior tumor targeting, whereas accumulation in the liver was diminished compared to non‐targeted liposomes. Moreover, FA‐targeting liposomes showed the greatest suppression of xenograft tumor growth compared to treatments with free drugs or non‐targeted liposomes formulations.^[^ ^200^ ^]^

In addition to decorating the surface of EVs with FA, other small molecules have been studied to improve active targeting. Macrophage‐derived PTX‐loaded EVs conjugated with aminoethylanisamide‐polyethylene glycol (AA‐PEG) colocalized significantly more with lung metastases, indicating efficient targeting of EVs in vivo. Noteworthy, AA‐PEG‐conjugated EVs were not found in the lungs of healthy animals. Furthermore, targeting of EVs demonstrated improved therapeutic outcomes upon intravenous administration, as compared to non‐targeted EVs.^[^ ^140^ ^]^ In another study, EVs were targeted toward hepatocyte asialoglycoprotein receptors by modifying the surface with cationized pullulan, a maltotriose consisting of three glucose moieties. In a mouse liver injury model, despite an increased accumulation of targeted EVs in the liver, the vast majority of EVs accumulated in the lungs and in the spleen upon intravenous injection.^[^ ^201^ ^]^ Similarly, the surface of liposomes has been engineered with small molecules to improve accumulation of liposomes at specific sites, such as, the brain. For example, Salem et al. functionalized nanoliposomes with escalating glutathione‐maleimide‐PEG_2000_‐distearoyl phosphatidylethanolamine (glutathione‐moiety) mole percent ranging from 0 to 0.75 mol%, to enhance the targeting of flucytosine to the brain. According to the ratios of the flucytosine concentration in the brain versus its concentration in plasma, increasing mol% of glutathione‐moiety results in significantly enhanced flucytosine targeting in the brain. Compared to other organs, the targeting efficiency of flucytosine‐loaded 0.75% mole/mole glutathione‐modulated liposomes compared to 0% mole/mole glutathione‐engineered liposomes was most pronounced in the brain, as determined by concentration efficiency, drug‐targeting index, and relative efficiency.^[^ ^202^ ^]^ Overall, these results revealed that modifying the surface of liposomes with a glutathione‐moiety enhanced the delivery of flucytosine to the brain.

### 7.2 DNA and RNA Aptamers

Aptamers are defined as short, synthetic and single‐stranded DNA or RNA molecules that are able to bind to their target with high affinity and specificity. Nucleic aptamers are characterized by unique features, such as small size, simple synthesis process, low immunogenicity, high affinity and target selectivity, and stability under a wide variety of physiochemical conditions.^[^ ^203^ ^]^ Generally, aptamers are added onto EVs and liposomes through physical or chemical methods, usually by covalently linking terminally altered aptamers to lipid chains or PEG molecules. In liposomes, non‐covalent linkage of aptamers is usually achieved by electrostatic coupling of negatively charged aptamers to positively charged liposomes.^[^ ^203^ ^]^ Wan and coworkers used the nucleolin‐targeting DNA aptamer AS1411 to target the delivery of EVs carrying PTX toward MDA‐MB‐231 tumor xenograft in mice.^[^ ^204^ ^]^ The aptamer was covalently conjugated to cholesterol‐PEG and incorporated into the membrane of mouse DCs. Subsequently, these cells were mechanically extruded to obtain aptamer‐targeted CDNs. Biodistribution of intravenously administered CDNs was studied by determining the concentration of PTX in different tissue samples after treatment. After 12 h, PTX concentrations were highest in liver and spleen, suggesting that, despite active targeting, CDNs accumulate at higher levels in the liver and spleen compared to tumors. However, compared to free PTX and PTX encapsulated in unmodified EVs, PTX‐loaded AS1411‐CDNs showed the highest accumulation in tumors and demonstrated the strongest anti‐tumor activity.^[^ ^204^ ^]^ The same DNA aptamer was used to target miRNA let‐7 encapsulated EVs toward breast cancer tumors in mice. Interestingly, labeling the EVs with Cy5 showed that AS1411‐EVs accumulated preferably in tumor tissue, whereas other organs, such as liver and spleen, showed weak fluorescence signals 4.5 h after intravenous administration.^[^ ^205^ ^]^ In a more recent study, a valency‐controlled tetrahedral DNA nanostructure conjugated with three cholesterol anchors and one TLS11a aptamers was designed to target EVs toward HepG2 xenograft tumors in mice. Targeted EVs loaded with CRISPR‐Cas9 RNA‐guided endonucleases (RNP) showed termination of tumor development, whereas unmodified EVs loaded with RNP did not significantly suppress tumor development. An in vivo biodistribution study showed that TLS11a‐EVs demonstrated higher accumulation in tumor tissue compared to unmodified EVs, whereas targeting EVs with TLS11a aptamers resulted in decreased liver and kidney accumulation.^[^ ^206^ ^]^ In another study, a three‐way junction was used as a building block to fuse cholesterol and a RNA aptamer targeting prostate‐specific membrane antigen (PSMA). PSMA aptamer‐displaying EVs were loaded with survivin siRNA and significantly inhibited prostate cancer xenograft in mice.^[^ ^207^ ^]^ Unfortunately, this study did not investigate the in vivo biodistribution of EVs decorated with PSMA aptamers.

In addition to EVs being decorated with nucleic aptamers, several aptamers have been conjugated to liposomes to enhance pharmacokinetic behavior in vivo, as has been extensively reviewed by Moosaviana and Sahebkar.^[^ ^203^ ^]^ The nucleolin targeting aptamer AS1411 was conjugated to PEGylated cationic liposomes loaded with anti‐BRAF siRNA (siBRAF) to target A375 tumor xenografts in mice. After 48 h, tumors from mice treated with AS1411‐targeted liposomes possessed elevated signals, whereas the highest fluorescence signals were observed in the kidneys from all groups, suggesting renal excretion.^[^ ^208^ ^]^ Regrettably, ex vivo analysis of AS1411‐targeted liposome biodistribution at an earlier time point was not studied, whereas in vivo real‐time imaging clearly showed substantial accumulation of liposomes in the liver at earlier time points.

In general, active targeting of both liposomes and EVs using AS14411‐aptamer results in increased therapeutic activity, whereas it is not yet clear if vesicles preferentially accumulate at the intended site of targeting. Furthermore, the clinical application of aptamers as targeting moieties might be limited due to possible loss of targeting capacity under in vivo conditions.^[^ ^209^ ^]^ Ding et al. investigated the loss of targeting capacity by examining how the physiological milieu affected targeting effect of aptamer functionalized gold nanoparticles. Results demonstrated that targeting ability loss was caused by protein corona blocking, replacement, and enzymatic cleavage of surface aptamer targeting ligands.^[^ ^209^ ^]^

### 7.3 pH‐Responsive Targeting

Another targeting strategy uses unique characteristics of target‐tissue and tumors. For instance, the tumor microenvironment utilizes, unlike normal cells, the energy produced from oxygen‐independent glycolysis for survival resulting in large amounts of lactate, which contributes to enhanced acidification of the extracellular tumor microenvironment.^[^ ^210^ ^]^ pH‐responsive DDS are a promising strategy to target tumors and deliver anti‐tumor drugs. For example, Lee and colleagues engineered pH‐responsive EVs containing hyaluronic acid grafted with 3‐(diethylamino)propylamine (HDEA) loaded with DOX.^[^ ^211^ ^]^ Compared to free Ce6 dye and pH‐insensitive modified EVs, HDEA‐engineered EVs demonstrated the highest accumulation in tumors, whereas accumulation in the liver or other clearance organs was remarkably lower. In addition, modifying EVs to be pH responsive resulted in enhanced anti‐tumor activity.^[^ ^211^ ^]^ Similarly, Chiang and coworkers designed pH‐responsive polymer liposomes targeting the tumor ECM composed of DMPC, methoxy‐PEG‐*b*‐poly(*N*‐2‐hydroxypropyl methacrylamide‐*co*‐histidine‐cholesterol polymers, and biotin‐PEG‐biotin (biotin) crosslinkers.^[^ ^212^ ^]^ Biodistribution studies were carried out in Balb‐C/nude mice and three treatments were used: Cy5.5‐labeled Dox‐loaded polymer‐incorporated liposomes (PILs), Cy5.5‐labeled Dox‐loaded ECM‐targeting liposomes (ECMLs), and Cy5.5‐labeled Dox‐loaded ECMLs combined with excess of free biotin molecules (ECMLs + biotin). Compared to PILs, ECMLs and ECMLs + biotin treatments resulted in the highest tumor accumulation, with a significantly weaker fluorescence signal in other organs 24 h post‐intravenous injection. In addition, in their work, because ECMLs accumulated preferably in tumor, ECMLs demonstrated superior anti‐tumor activity in vivo.^[^ ^212^ ^]^

### 7.4 Magnetic Targeting

Both liposomes and EVs have been actively targeted toward diseased tissue without the incorporation of targeting ligands, but instead by the addition of magnetic particles. Enhanced drug delivery at the target site can be accomplished by application of an external magnetic field gradient. The most common method for incorporating magnetic particles in liposomes is thin film hydration followed by sonication and extrusion or by reversed phase evaporation. Subsequently, magnetic particles in an aqueous fluid are incorporated into the liposomes upon hydration of the lipid film.^[^ ^213^ ^]^ On the other hand, magnetic‐responsive EVs are most commonly generated through pre‐conditioning parent cells with magnetic particles or by post‐isolation modification through chemical engineering.^[^ ^214^ ^]^

For instance, Silva and colleagues loaded human macrophages with iron oxide nanoparticles and different therapeutic agents: DOX, t‐PA, and two photosensitizers, resulting in magnetic‐responsive EVs. Upon applying a magnetic field, modified EVs demonstrated enhanced drug uptake by cancer cells and increased cytotoxicity in vitro compared to unmodified EVs.^[^ ^214a^ ^]^ Another example was reported by Qi and coworkers, who designed a dual‐functional EV‐based superparamagnetic nanoparticles cluster as a targeted drug delivery vehicle for cancer therapy.^[^ ^214b^ ^]^ Upon intravenous administration in mice bearing subcutaneous H22 cancer, EVs with magnetic‐targeting ability delivered DOX to cancer cells and inhibited tumor growth significantly. Furthermore, biodistribution studies revealed the highest fluorescence signal in tumors to which a magnetic field had been applied, whereas the absence of a magnetic field resulted in decreased accumulation in the tumor and enhanced accumulation in the liver.^[^ ^214b^ ^]^ More recently, BAY55‐9837, a potential therapeutic peptide against type 2 diabetes mellitus, was encapsulated into EVs that were coupled with superparamagnetic iron oxide nanoparticles.^[^ ^215^ ^]^ In vivo biodistribution studies showed that BAY55‐9837‐loaded EVs enhanced accumulation in the pancreas under an external magnetic field, whereas accumulation in the liver was lower compared to EVs administered in the absence of a magnetic field.^[^ ^215^ ^]^

Similarly, magnetic liposomes have been engineered to carry drugs toward the targeted tissue, such as tumor tissue, by applying an external magnetic field gradient. For instance, Lin and colleagues designed magnetic hyperthermia‐sensitive liposomes by combining magnetic fluid Fe_3_O_4_ and thermosensitive lipids, for the delivery of DOX in a MCF‐7 xenograft murine model.^[^ ^216^ ^]^ 12 h after intravenous administration, the strongest fluorescence signals were observed in tumors under a magnetic field, whereas less or no fluorescence was reported in other isolated organs. Furthermore, superior anti‐tumor efficacy was observed in mice treated with magnetic liposomes under an external magnetic field, compared to treatment groups treated with PBS, free DOX, and in the absence of a magnetic field gradient.^[^ ^216^ ^]^ In another example, liver‐targeted gene delivery was studied using magnetic cationic liposomes holding a luciferase reporter gene‐containing plasmid DNA guided by a magnetic field.^[^ ^217^ ^]^ In vivo transfection studies demonstrated higher expression of the reporter gene in the liver when the liver of rats was treated with a magnetic force. Interestingly, highest transfection effectivity was reported in the lung and spleen, suggesting off‐target accumulation of the magnetic cationic liposomes under magnetic guidance.^[^ ^217^ ^]^ Li et al. designed a novel dual‐targeting nanocarrier by incorporating octreotide‐peptide and magnetic Fe_3_O_4_ nanoparticles into the surface of oleanolic acid‐loaded liposomes.^[^ ^218^ ^]^ In vivo biodistribution studies were performed in somatostatin receptor 2 positive S180 tumor‐bearing mice and showed accumulation in the tumor, compared to free oleanolic acid, untargeted liposomes, and targeted liposomes in the absence of a magnetic field. In addition, upon magnetic field, dual‐targeted liposomes demonstrated a more effective anti‐tumor treatment.^[^ ^218^ ^]^

Altogether, these pilot studies lay the foundation for using magnetic‐sensitive liposomes or EVs as targeted DDS. Several biodistribution studies show that magnetic guidance of magnetic‐dependent targeted liposomes and EVs exhibit similar accumulation patterns, suggesting that the RES/MPS can be, at least to some extent, circumvented. However, more research needs to be conducted on difficulties targeting deep tissues in the body using an external magnetic field. Furthermore, toxicity issues may arise with the use of iron‐based nanoparticles.

## 8 Comparison of Liposomes and Extracellular Vesicles for Drug and Nucleic Acid Delivery

### 8.1 Small Molecule Delivery via Vesicles

An increasing number of studies is emerging that performs a head‐to‐head comparison of liposomes and EVs. For example, for the delivery of small molecules. Millard et al. used HUVEC (human umbilical vascular endothelial cell) EVs as delivery vehicles for a photosensitizing agent and compared these with a commercial liposomal formulation, based on DPPC and DPPG in a ratio of 9:1.^[^ ^219^ ^]^ Uptake of EVs in HT26 cells was shown to be more efficient than uptake of the liposomes in a 24‐h timeframe. In a 3D spheroid model, EVs were found to penetrate deeper into the core than the liposomes could. Schindler et al. studied uptake of vesicle formulations carrying DOX.^[^ ^220^ ^]^ Myocet and Doxil, two commercially available liposomes, were compared to an undisclosed control liposomal formulation and HEK293 EVs. Uptake in HEK293 cells was significantly more effective for HEK293 EVs than for the liposomes used, which all had comparable uptake profiles. Studies on efficacy in a panel of cell lines showed that the EV formulation gave by far the lowest IC50. Heusermann et al. compared uptake of EVs to that of a liposomal formulation composed of cationic lipid, cholesterol, and a PEG‐conjugated lipid in a ratio of 50:46:4.^[^ ^221^ ^]^ The liposomes were found to accumulate at the surface of HEK293 as islands that grew over time. Only a minor fraction was shown to be taken up in the cell after a few hours. Conversely, EVs did not accumulate at the cell surface and entered the cells as single vesicles within minutes of addition. Overall, these results suggest that EVs may be taken up more efficiently into target cells than the liposomal formulation used. This may translate into an enhanced delivery of the cargo contained within EVs. Sun and coworkers compared delivery of curcumin by EVs with its delivery by liposomes from a commercial source to lipopolysaccharide‐challenged mice.^[^ ^222^ ^]^ Mice receiving EVs had a lower mortality than mice administered liposomes with an equivalent concentration of curcumin, indicating that EVs may be more efficient in this setting to bring the therapeutic to its target or that EVs may encompass innate therapeutic effects.

Jang et al. compared the antitumor activity of Raw264.7 macrophage CDNs loaded with DOX, to DOX included within HSPC:DSPE‐PEG_2000_:cholesterol (54:2:44) liposomes.^[^ ^223^ ^]^ In a tumor‐bearing mouse model, the drug‐loaded CDNs were significantly more effective in suppressing tumor growth, than the liposomes and even as free drug in a similar concentration. Comparison with CDNs and EVs derived from U937 lymphoma cells, revealed similar efficacy to the Raw264.7 CDNs. After treatment of the CDNs with trypsin, to digest the membrane proteins, the Raw264.7 CDN‐suppression was reduced to the level seen for the liposomes. In contrast, when Smyth et al. examined the efficacy of DOX‐loaded vesicles in a 4T1 tumor‐bearing mouse model, free DOX performed similar to the vesicle formulations used.^[^ ^43^ ^]^ 4T1 EVs were compared to phosphatidylcholine:cholesterol liposomes with a mole percentage of 67:33, and to an EV‐inspired liposome formulation, consisting of phosphatidylcholine:phosphatidylethanolamine:phosphatidylserine:sphingomyelin:cholesterol at a mole percentage of 21:17.5:14:17.5:30. Of these formulations phosphatidylcholine:cholesterol was slightly less effective than EVs and the EV‐inspired liposome formulation. These results would encourage the further exploration of the latter.

### 8.2 Nucleic Acid Delivery Using Vesicles

For the delivery of nucleic acids, cationic liposome formulations have been a commonly used method. However, from in vitro cell cultures it is recognized that some cell types are more refractory to transfection in this manner than other cell types. The in vivo application of these formulations is also limited, as their stability in the bloodstream is low, the delivery of nucleic acids into target cells is often weak, and toxicity is an issue.^[^ ^224^ ^]^ As such, the development of improved methods by which nucleic acids, such as siRNAs can be delivered is highly desired. Furthermore, a delivery system that also has potential for translation to the in vivo setting would be advantageous. In this context, EVs have been proposed as a delivery system with potential benefits worth exploring.

Singh et al. showed that human airway epithelial cells were susceptible to delivery of siRNA with EVs derived from A549 cells.^[^ ^225^ ^]^ A knockdown efficiency of ≈50% could be achieved for a chosen model gene (hypoxanthine phosphoribosyltransferase). Although a direct comparison with liposome‐based strategies was not made, this cell type is known to be difficult to transfect with standard techniques. Lamichhane et al. loaded HEK293T‐derived EVs with siRNA against GAPDH (glyceraldehyde 3‐phosphate dehydrogenase) using a sonication approach. They showed that exposure of HEK293T cells to EVs with increasing siRNA concentrations led to a dose‐dependent reduction in GAPDH expression.^[^ ^226^ ^]^ However, a commercial transfection reagent, based on a mixture of cationic and neutral lipids, was more efficient in comparison with EVs. Alvarez‐Erviti et al. used EVs to deliver siRNA, both in vitro and in vivo to the brain in mice.^[^ ^227^ ^]^ Crossing the blood‐brain barrier is an endeavor that is challenging for synthetic nanoparticles. Here, dendritic cells were engineered to express the Lamp2b protein fused to RVG, a neuron‐targeting peptide, and loaded with siRNA after EV purification. In vitro, silencing of the GAPDH and BACE1 (beta‐secretase 1)‐gene in Neuro2A cells was equally effective using RVG‐EVs or Lipofectamine 2000, a commercial cationic liposome‐based transfection reagent. However, in a mouse model, RVG‐EVs were far more effective at silencing BACE1, than cationic liposomes.^[^ ^227^ ^]^ These results indicate that in vivo, targeted EVs may be advantageous for siRNA delivery in comparison with synthetic liposomes. However, unmodified EVs were ineffective for delivery both in vitro and in vivo. It should be noted that RVG‐targeted liposomes were not explored in this study. Without a head‐to‐head comparison, it is challenging to assess whether EVs or liposomes would be preferred in this context. Recently, Dos Santos Rodrigues et al. explored the addition of RVG peptides on pegylated liposomes, consisting of DOPE:DOTAP:cholesterol:RVG‐PEG_2000_‐DSPE (45:45:2:4 mol%).^[^ ^188^ ^]^ The authors showed that these had superior ability to transfect cells compared to non‐functionalized liposomes and also that they were able to distribute to the brain in a mouse model. However, in line with earlier observations, despite the functionalization liver and kidneys remained the main sites of accumulation.

The relative efficacy of EVs versus liposomes is likely to be dependent on the cell or tissue type that is being targeted, as well as on the composition of the liposomes used. EVs carrying siRNA or shRNA against KRAS^G12D^, were shown to lower the mRNA levels of KRAS^G12D^ in pancreatic cancer cells more efficiently than liposomes loaded with similar levels of RNAi molecules. Similarly, these EVs were better able to suppress growth of orthotopic xenografted pancreatic tumors in mice compared to liposomes.^[^ ^46^ ^]^ Stremersch et al. investigated the delivery of cholesterol‐siRNA conjugates via EVs and liposomes.^[^ ^228^ ^]^ Liposomes were composed of a 6:4 mixture of DOPE:CHEMS. Though equal particle concentrations and siRNA loading were compared, only the liposomes showed functional siRNA delivery to cells with significant downregulation of target gene expression, not the EVs. This would indicate that also in this particular set‐up, native EVs were less effective than synthetic liposomes in delivering their cargo.

Likely, cellular interaction, route of uptake and intracellular processing are factors influencing outcome. To come to the most optimal system for RNA delivery in the vivo situation, extensive knowledge is necessary in all these areas. In this respect, lipid composition and the potential necessity to functionalize the delivery vehicles used are important avenues that warrant further analysis. Currently, the designs of lipid formulations which mimic EV compositions to enhance cellular uptake efficiency of liposomes are being explored. EV‐like lipid formulations composed of DOPC:sphingomyelin:cholesterol:DOPS:DOPE (21:17.5:30:14:17.5 mol%) were investigated for the delivery of siRNA to cells and compared to various other liposome formulations.^[^ ^229^ ^]^ Results indicated the EV‐mimics carried a net negative surface charge. Compared to near neutral phosphatidylcholine:cholesterol liposomes at a molar ratio of 70:30, the EV‐mimics showed a higher cellular uptake. Uptake was still inferior to positively charged DOTAP liposomes, formulated with DOTAP:DOPC:cholesterol at a molar ratio of 40:40:20. The cellular internalization mechanisms of the EV‐inspired lipid formulation resemble those of EVs, namely, caveolae‐mediated endocytosis, macropinocytosis and membrane fusion. The latter appears to be cell type dependent and could be exploited for lysosomal escape.^[^ ^229^ ^]^ In comparison with the DOTAP liposomes and the commercial Lipofectamine 2000 reagent, silencing of VEGF via siRNA was less effective with the EV‐inspired formulation. This was suggested to be a consequence of the higher encapsulation efficiency in cationic liposomes, though the reduced uptake could also play a role.^[^ ^229^ ^]^ An alternative strategy that is being explored is the direct fusion between liposomes and EVs, as a means to tailor lipid composition, but also to enable loading of contents into the vesicles.^[^ ^230^ ^]^ This approach was shown to be successful for the delivery of large plasmids into MSCs.^[^ ^230^ ^]^ Where liposomes alone and HEK293FT‐EVs alone were unable to carry this cargo into the target cells, delivery of the plasmid by the hybrid between these two showed significant expression levels. The liposomal formulation used for this was commercial Lipofectamine 2000. Treatment of the hybrid formulation with proteinase K abrogated the observed effects, indicating the importance of the presence of the biological entities.^[^ ^230^ ^]^

Overall, the assessment of whether EVs are an efficient moiety for delivery of nucleic acids shows variable results. The limited consensus may indicate that the context of the experiments is crucial. Therefore, more in depth analysis and comparison of the different vesicle types is necessary for a thorough evaluation of efficacy.

## 9 Conclusion and Future Perspectives

Overall, we consider both liposomes and EVs to have innate advantages and disadvantages, when it comes to their characteristics as drug delivery entities. Due to their biological origin and therefore their anticipated biocompatibility, the in‐depth analysis of EVs as drug delivery entities may generate valuable insights that can be applied to synthetic particles. Examples are the tailoring of the lipid composition or functionalization with various bioinspired moieties to generate next‐generation liposomal drug delivery vehicles with enhanced efficiency and biocompatibility. Insights from the EV‐field could also be advantageous for the homing of vesicles to target tissue as well as optimal uptake in target cells and efficient delivery of therapeutic cargo. On the other hand, the work on liposomes has had a head start on the EV‐field. As a consequence, multiple methodologies and technologies are available that could be applied to the characterization and engineering of EVs instead. For example, in terms of the pharmacokinetic properties of liposomes and EVs, the generalized picture that emerges is that both vesicle types are cleared rapidly from the bloodstream and that the liver and/or spleen are the main sites of accumulation in the body. The strategies that have been developed in the liposome field over the years to circumvent these biological barriers, may also help refining EVs. Furthermore, the progress that has been made on large scale production and drug loading strategies of liposomes could potentially be exploited. Liposomes are manufactured from the bottom up, offering extensive control over their composition. This is very different for EVs, which are secreted from cells. As elucidation of the underlying processes by which cells select cargo for incorporation into vesicles is still ongoing, we have only limited control over the composition of EVs. Nonetheless, strategies are emerging through which EV content can be influenced, for example, by modification of the parent cells through genetic means or culture conditions, or by engineering EVs after purification.

**Figure 6:**
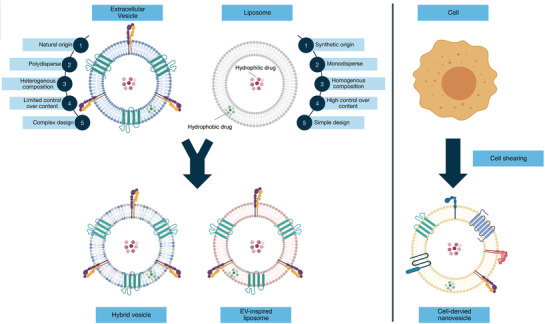
Overview of next‐generation vesicle formulations for DDS. Comparing EVs and liposomes on various aspects (no. 1–5), shows that they have inherent advantages and disadvantages. The generation of EV‐inspired liposomes or EV/liposome hybrid vesicles could be key to improving lipid vesicles as a DDS as they could incorporate the beneficial aspects of both EVs and liposomes. Another alternative for the generation of vesicles of biological origin, is to use the cellular membranes in a cell shearing approach.

Currently, it is recognized that some clinically used liposome‐encapsulated drugs, rather than substantially improving drug efficacy, are more effective at reducing toxicity. Furthermore, liposomes may function as a reservoir for more continuous drug release, which also reduces toxicity. Whether this is also valid for EVs or whether additional targeting can be achieved by these vesicles remains to be established, though initial observations are promising. The number of studies reporting on a head‐to‐head comparison between liposomes and EVs is still limited. However, it would appear that the overall pharmacokinetic profiles of EVs and liposomes are very similar, though there are also studies reporting advantageous properties for EVs. Both vesicle types have their own advantages with liposomes offering extensive control over contents and EVs having the advantage of inherent biocompatibility and a complex biological composition that will be challenging to fully recapitulate in liposomes. Combined they could advance vesicles as drug delivery entities. Three main strategies have emerged to integrate the best of both worlds, merging the advantageous properties of liposomes with those of EVs. An overview of these approaches is provided in **Figure** **6**.

The first strategy is to generate hybrid vesicles, consisting of both EV and liposomal components.^[^ ^231^ ^]^ This confers the tunability of synthetic liposomes onto natural EVs and enables modification of the EV lipid bilayer. This may tune the cellular uptake of the vesicle and could aid in enhancing EV stability, circulation time, targeting properties, etc. Second, the inherent biocompatibility of EVs serves as an inspiration for the liposome‐field to generate synthetic vesicles that incorporate the advantageous characteristics of natural vesicles. As such, the lipid compositions of liposomes are becoming increasingly complex in an effort to resemble the lipidomic characterization of EVs. Furthermore, the addition of targeting moieties to the liposome surface takes inspiration from findings of EVs. This type of bioinspired approach, which retains the extensive control over the content of the vesicles generated, could be highly advantageous for future clinical approval. The challenge before us is identifying the lipids and proteins exposed on the EV surface that are critical for successful delivery of drugs to the various sites we want to target, whilst minimizing interaction with undesired targets. A third approach that is being explored, is the generation of vesicles from cellular membranes using a cell shearing procedure.^[^ ^62^, ^223^ ^]^ This method is aimed at retaining the biocompatibility of vesicles, whilst increasing the scale of production and increasing the control over internal cargo. Expression of the appropriate membrane markers on the cells, for example by taking inspiration from EVs, could be highly advantageous for the generation of vesicles for targeted drug delivery.

## Conflict of Interest

The authors declare no conflict of interest.

## Supporting information

Supporting Information
