# Supplementary material for: Liposomes and Extracellular Vesicles as Drug Delivery Systems: A Comparison of Composition, Pharmacokinetics, and Functionalization
Source: Adv Healthc Mater. 2021 Jun 24;11(5):2100639. doi: 10.1002/adhm.202100639 (PMC11468589; doi:10.1002/adhm.202100639)
Supplement: Supplementary file 1 — Supporting Information [file ADHM-11-2100639-s001.pdf]

WILEY-VCH

© 2021 Wiley-VCH GmbH

ADVANCED HEALTHCARE MATERIALS

Supporting Information

for *Adv. Healthcare Mater.*, DOI: 10.1002/adhm.202100639

Liposomes and Extracellular Vesicles as Drug Delivery Systems: A Comparison of Composition, Pharmacokinetics, and Functionalization

*Luke van der Koog, Timea B. Gandek and Anika Nagelkerke**

Supporting Information

Liposomes and Extracellular Vesicles as Drug Delivery Systems: A Comparison of Composition, Pharmacokinetics, and Functionalization.

Luke van der Koog, Timea B. Gandek and Anika Nagelkerke[*]

Supplementary Table 1. Overview of lipids and lipid-like components available for the generation of liposomes. Synthetic analogues are also included. Charge is at neutral pH.

**Charge at neutral pH**	**Abbreviation**	**Name in full**	**Number of carbons : Number of double bonds**
Cationic	DDAB	Dimethyldioctadecylammonium	18 : 0
	DODMA	1,2-dioleyloxy-3-dimethylaminopropane	18 : 1
	DOTMA	1,2-di-O-octadecenyl-3-trimethylammonium propane	18 : 1
	DODAP	1,2-dioleoyl-3-dimethylammonium-propane	18 : 1
	DOTAP	1,2-dioleoyl-3-trimethylammonium-propane	18 : 1
Anionic	PI	Phosphatidylinositol	[a)]
	PS	Phosphatidylserine	[a)]
	DLPS	1,2-dilauroyl-*sn*-glycero-3-phospho-L-serine	12 : 0
	DMPS	1,2-dimyristoyl-*sn*-glycero-3-phospho-L-serine	14 : 0
	DPPS	1,2-dipalmitoyl-*sn*-glycero-3-phospho-L-serine	16 : 0
	DSPS	1,2-distearoyl-*sn*-glycero-3-phospho-L-serine	18 : 0
	DOPS	1,2-dioleoyl-*sn*-glycero-3-phospho-L-serine	18 : 1
	PG	Phosphatidylglycerol	[a)]
	DLPG	1,2-dilauroyl-*sn*-glycero-3-phosphoglycerol	12 : 0
	DMPG	1,2-dimyristoyl-*sn*-glycero-3-phosphoglycerol	14 : 0
	DPPG	1,2-dipalmitoyl-*sn*-glycero-3-phosphoglycerol	16 : 0
	DSPG	1,2-distearoyl-*sn*-glycero-3-phosphoglycerol	18 : 0
	DOPG	1,2-dioleoyl-*sn*-glycero-3-phosphoglycerol	18 : 1
	DLPA	1,2-dilauroyl-*sn*-glycero-3-phosphatidic acid	12 : 0
	DMPA	1,2-dimyristoyl-*sn*-glycero-3-phosphatidic acid	14 : 0
	DPPA	1,2-dipalmitoyl-*sn*-glycero-3-phosphatidic acid	16 : 0
	DSPA	1,2-distearoyl-*sn*-glycero-3-phosphatidic acid	18 : 0
	DOPA	1,2-dioleoyl-*sn*-glycero-3-phosphatidic acid	18 : 1
Zwitterionic	PC	Phosphatidylcholine	[a)]
	DLPC	1,2-dilauroyl-*sn*-glycero-3-phosphocholine	12 : 0
	DMPC	1,2-dimyristoyl-*sn*-glycero-3-phosphocholine	14 : 0
	DPPC	1,2-dipalmitoyl-*sn*-glycero-3-phosphocholine	16 : 0
	DSPC	1,2-distearoyl-*sn*-glycero-3-phosphocholine	18 : 0
	DOPC	1,2-dioleoyl-*sn*-glycero-3-phosphocholine	18 : 1 (Δ9-Cis)
	POPC	1-palmitoyl-2-oleoyl-*sn*-glycero-3-phosphocholine	16 : 0 - 18 : 1

SOPC	1-stearoyl-2-oleoyl-*sn*-glycero-3-phosphocholine	18 : 0 - 18 : 1
PE	Phosphatidylethanolamine	[a]
DLPE	1,2-dilauroyl-*sn*-glycero-3-phosphorylethanolamine	12 : 0
DMPE	1,2-dimyristoyl-*sn*-glycero-3-phosphorylethanolamine	14 : 0
DSPE	1,2-distearoyl-*sn*-glycero-3-phosphorylethanolamine	16 : 0
DPPE	1,2-dipalmitoyl-*sn*-glycero-3-phosphorylethanolamine	18 : 0
DOPE	1,2-dioleoyl-*sn*-glycero-3-phosphorylethanolamine	18 : 1

[a] From natural sources, lipids typically come in a mixture and cannot be presented by a single carbon and saturation number.

Supplementary Table 2. Overview of lipidomic analyses performed on EVs from in vitro sources.

Source of EVs	Isolation method	Lipid composition	Ref
Primary healthy colon cells and four colon cancer cell lines - HT29, SW480, and LS174t (from primary site), and Colo 201 (from metastatic site).	UC	Most abundant membrane lipids in EVs: phosphatidylcholine and sphingomyelin. ↑ sphingomyelin in EVs compared to parent cells. Ceramide ↑ or maintained depending on EV/cell type. Other lipids assessed (PE, PE plasmalogens, PS, and PI), levels ↓ or maintained depending on EV/cell type.	[1]

	Primary		HT29		SW480	
	**Cells**	**EVs**	**Cells**	**EVs**	**Cells**	**EVs**
**PC:**	44.9%	29.8%	50.4%	61.6%	53.6%	60.3%
**SM:**	11.1%	34.8%	7.2%	34.0%	6.0%	34%
**Cer:**	1.4%	3.4%	1.8%	1.0%	1.5%	0.8%
**PE:**	25.1%	10.3%	8.2%	0.9%	10.8%	1.3%
**PE P-:**	5.4%	6.3%	18.5%	0.9%	13.4%	1.2%
**PI:**	4.8%	4.1%	7.5%	0.4%	6.4%	1.1%
**PS:**	7.4%	11.3%	6.4%	1.4%	8.4%	1.3%

	LS174t		Colo 201	
	**Cells**	**EVs**	**Cells**	**EVs**
**PC:**	49.2%	58.4%	53.6%	48.6%
**SM:**	9.0%	35.4%	7.8%	28.4%
**Cer:**	1.2%	1.3%	0.6%	0.6%
**PE:**	11.7%	1.3%	11.6%	3.3%
**PE P-:**	14.8%	1.1%	13.9%	7.9%

<table>
<tr><td></td><td></td><td><table><tr><td>PI:</td><td>5.6%</td><td>0.6%</td><td>5.4%</td><td>0.9%</td></tr><tr><td>PS:</td><td>8.5%</td><td>1.9%</td><td>7.1%</td><td>10.3%</td></tr></table></td><td></td></tr>
<tr><td>RBL-2H3 - rat mast cells and human dendritic cells.</td><td>UC</td><td>↑ in sphingomyelin and disaturated molecular species (e.g. phosphatidylethanolamines). No change in cholesterol and lyso(bis)phosphatidic acid. ↓ in phosphatidylcholine.</td><td>[2]</td></tr>
<tr><td>U87 glioblastoma cells, Huh7 hepatocellular carcinoma cells and human bone marrow-derived MSCs.</td><td>UC</td><td>Differential UC protocol to enrich a population of microvesicles and exosomes. Key findings:
· MSC and Huh7 exosomes similar lipid profile.
· All MVs ↑ ceramides and sphingomyelins.
· U87 exosomes ↑ in sphingomyelins.
· MSC and U87 MVs, and U87 exosomes ↑ in zwitterionic lipid head groups (phosphatidylcholines and/or phosphatidylethanolamines), ↓ in other head groups.
· MSC and Huh7 exosomes and MSC MVs ↑ in long lipids (> 60 carbons) and polyunsaturated lipids (> 10 double bonds).
· MSC and Huh7 exosomes ↑ in fully saturated free fatty acids and cardiolipin.
· MSC and Huh7 MVs ↑ cholesterol esters.
· MSC MVs ↑ acyl carnitines and lysophosphatidylcholines.
· All exosomes ↑ glycolipid, free fatty acid and phosphatidylserine, ↓ or no change for MVs, except phosphatidylserine ↑ in U87 MVs.
· MSC and Huh7 exosomes ↑ lyso-derivatives of phosphatidylserines, phosphatidylglycerols and phosphatidylinositols. U87 exosomes ↑ lyso-phosphatidylethanolamines were rather enriched in U87 exosomes. These lyso-derivatives ↑ in MSC MVs ↓from U87 and Huh7 MVs.
· All exosomes and most MVs ↓ structural membrane lipids, including phosphatidylglycerols, phosphatidylinositols and phosphatidylethanolamines.
All exosomes ↓ phosphatidylcholines, no change or ↑ in MVs.</td><td>[3]</td></tr>
<tr><td>RWPE1 (non-tumourigenic), NB26 (tumourigenic) and PC-3 (metastatic) prostate cell lines.</td><td>UF</td><td>↓ in glycerolipids, ↑ in sphingolipids and glycerophospholipids in NB26 and PC-3 EVs compared to RWPE1 EVs.
<table><tr><th>EVs</th><th>RWPE1</th><th>NB26</th><th>PC-3</th></tr><tr><td>Glycerolipids</td><td>33%</td><td>28%</td><td>26%</td></tr><tr><td>Glycero-phospholipids</td><td>33%</td><td>36%</td><td>38%</td></tr><tr><td>Sphingolipids</td><td>27%</td><td>30%</td><td>28%</td></tr><tr><td>Cholesterol Esters</td><td>5%</td><td>4%</td><td>6%</td></tr><tr><td>Others</td><td>3%</td><td>3%</td><td>2%</td></tr></table></td><td>[4]</td></tr>
<tr><td>SKOV-3 (ovarian cancer cells) and HOSEPiC (ovarian surface epithelial cells)</td><td>UC</td><td>SKOV-3 EVs enriched in ganglioside, zymosteryl, lysophosphatidylinositol, lysophosphatidylcholines, acylcarnitine, lipopolysaccharides, lysylphosphatidylglycerol, cholesterol ester; lower levels of ceramide, digalactosyldiacylglycerol, phosphatidylserine, phosphatidylinositol, phosphatidylglycerol, sphingomyelin, phosphatidylethanolamines and diglycerides than HOSEPiC EVs.</td><td>[5]</td></tr>
</table>

Mouse 3T3-L1 adipocytes.	UC	↑ cholesterol in small EV population<br>↑ externalised phosphatidylserine in large EV population.	[6]

	Large EVs:	Small EVs:
**PC**	58%	58%
**DAG**	0.9%	1.4%
**GlyCer**	0.028%	0.025%
**Cer**	0.57%	0.35%
**SM**	24.2%	22.9%
**PS**	0.7%	2%
**PI**	4.5%	4%
**PE**	4.25%	7%
**LPC**	6.8%	5%

High lymph node-metastatic D3H2LN and low-metastatic D3H1 MDA-MB-231 cells.	UC	Cholesterol and sphingomyelin enriched in EVs compared to cells, phosphatidylcholine and phosphatidylethanolamine levels were lower. Phosphatidylglycerol and phosphatidic acid were below limit of detection. PE-P levels were higher in D3H2LN than D3H1 EVs, cholesterol levels were higher in D3H1 than D3H2LN EVs.	[7]
Mouse cortical collecting duct principal cell line	UC	EVs released from the apical membrane differ from those released from basolateral membrane. Apical: ↑ sphingomyelin; Basolateral: ↑ cardiolipins, ceramides, and other phospholipids.	[8]
PC-3 prostate cancer cells.	UC	EVs ↑ in glycosphingolipids, sphingomyelin, cholesterol, and phosphatidylserine. EVs ↑ saturated and ↓ monounsaturated fatty acids than cells.	[9]

Lipid class	Cells (%mol)	EVs (mol%)	Lipid class	Cells (%mol)	EVs (mol%)
**Chol**	19.25 ± 0.97	43.52 ± 3.97	**PA**	0.09 ± 0.02	0.16 ± 0.00
**SM**	6.87 ± 0.55	16.26 ± 1.11	**PI**	1.03 ± 0.10	0.13 ± 0.01
**PC**	49.06 ± 3.27	15.28 ± 1.39	**LacCer**	0.04 ± 0.00	0.12 ± 0.01
**PS**	5.54 ± 0.94	11.66 ± 0.69	**LPI**	0.04 ± 0.01	0.09 ± 0.05

**PE**	10.59 ± 20	5.78 ± 0.96	**LPE**	0.07 ± 0.01	0.09 ± 0.00
**PE O + PE P**	2.67 ± 0.46	3.27 ± 0.42	**CE**	0.21 ± 0.04	0.08 ± 0.06
**DAG**	1.00 ± 0.08	1.52 ± 0.26	**Gb3**	0.01 ± 0.00	0.02 ± 0.00
**PC O + PC P**	2.04 ± 0.19	0.81 ± 0.05	**GM1**	0.0158	0.0472
**HexCer**	0.20 ± 0.03	0.76 ± 0.04	**GM2**	0.0009	0.0014
**Cer**	0.24 ± 0.02	0.32 ± 0.02	**GM3**	0.0053	0.0201
**PG**	1.03 ± 0.10	0.17 ± 0.07	**GD1**	0.0095	0.0171

U937 monocytes.	UC, study also employed a cell shearing approach to generate CDNs.	PE comparable between cells and EVs, ↓ in CDNs. PC ↑ in EVs ↑↑ in CDNs. SM ↑ in EVs and cells.	[10]

Lipid class	Cells	EVs	CDNs
**PE**	47%	41%	23.6%
**PC**	8%	34%	62.5%
**SM**	24%	19%	7.4%
**LPC**	3%	2%	5%
**Cer**	11%	1%	0.6%
**PS**	5%	2%	0.3%
**Others**	2%	1%	0.6%

Abbreviations: CDN = Cell-Derived Nanoparticle; CE = Cholesteryl esters; Cer = Ceramide; Chol = Cholesterol; DAG = Diacylglycerol; EV = Extracellular Vesicle; Gb3 = Globotriasylceramide; GD1, GM1, GM2, GM3 = Gangliosides; GlyCer = Glycosylceramide; HexCer = Hexocylceramide; LacCer = Lactocylceramide; LPC = Lysophosphatidylcholine; LPE = Lysophosphatidylethanolamine; LPI = Lyso phosphatidylinositol; MSC = Mesenchymal Stem Cell; MV = Microvesicle; PA = Phosphatidic acid; PC = Phosphatidylcholine; PC O / PC P = Ether-linked phosphatidylcholine; PE = Phosphatidylethanolamine; PE O / PE P = Ether-linked phosphatidylethanolamine; PG = Phosphatidylglycerol; PI = Phosphatidylinositol; PS = Phosphatidylserine; SM = Sphingomyelin; UC = Ultracentrifugation; UF = Ultrafiltration.

References

[1] J. Bestard-Escalas, A. Maimo-Barcelo, D. H. Lopez, R. Reigada, F. Guardiola-Serrano, J. Ramos-Vivas, T. Hornemann, T. Okazaki, G. Barcelo-Coblijn, *Cancers (Basel)* **2020**, 12.

[2] K. Laulagnier, C. Motta, S. Hamdi, S. Roy, F. Fauvelle, J. F. Pageaux, T. Kobayashi, J. P. Salles, B. Perret, C. Bonnerot, M. Record, *Biochem J* **2004**, 380, 161.

[3] R. A. Haraszti, M. C. Didiot, E. Sapp, J. Leszyk, S. A. Shaffer, H. E. Rockwell, F. Gao, N. R. Narain, M. DiFiglia, M. A. Kiebish, N. Aronin, A. Khvorova, *J Extracell Vesicles* **2016**, 5, 32570.

[4] J. S. Brzozowski, H. Jankowski, D. R. Bond, S. B. McCague, B. R. Munro, M. J. Predebon, C. J. Scarlett, K. A. Skelding, J. Weidenhofer, *Lipids Health Dis* **2018**, 17, 211.

[5] L. Cheng, K. Zhang, Y. Qing, D. Li, M. Cui, P. Jin, T. Xu, *J Ovarian Res* **2020**, 13, 9.

[6] M. Durcin, A. Fleury, E. Taillebois, G. Hilairet, Z. Krupova, C. Henry, S. Truchet, M. Trotzmuller, H. Kofeler, G. Mabilleau, O. Hue, R. Andriantsitohaina, P. Martin, S. Le Lay, *J Extracell Vesicles* **2017**, 6, 1305677.

[7] N. Nishida-Aoki, Y. Izumi, H. Takeda, M. Takahashi, T. Ochiya, T. Bamba, *Metabolites* **2020**, 10.
[8] V. D. Dang, K. K. Jella, R. R. T. Ragheb, N. D. Denslow, A. A. Alli, *FASEB J* **2017**, 31, 5399.
[9] A. Llorente, T. Skotland, T. Sylvanne, D. Kauhanen, T. Rog, A. Orlowski, I. Vattulainen, K. Ekroos, K. Sandvig, *Biochim Biophys Acta* **2013**, 1831, 1302.
[10] W. J. Goh, S. Zou, W. Y. Ong, F. Torta, A. F. Alexandra, R. M. Schiffelers, G. Storm, J. W. Wang, B. Czarny, G. Pastorin, *Sci Rep* **2017**, 7, 14322.

Supplementary Table 3. Overview of biodistribution of liposomes and EVs.

Type of particle	Isolation / Production method	Modification for visualization / analysis	Model	Injection	Dose	Strategy to alter biodistribution	Distribution	Ref
DOPE:DOTAP:Cholesterol:RVG-PEG_{2000}-DSPE (45:45:2:4) liposomes.	Thin lipid film hydration.	Fluorescently labelled with lissamine rhodamine-phosphatidylethanolamine.	Male and female C57BL/6 mice.	i.v.	~15.2 µmoles phospholipid/kg body weight.	Addition of RVG moiety and RVG-Tf moiety.	24 hours after injection: Liver and kidney mainly, followed by brain and spleen.	[1]
SL-HS (HSPC:SPC:CH: PEG-DSPE (12.5:37.5:40:5))<br>NGR-SL-HS (HSPC:SPC:CH: PEG-DSPE:NGR-PEG-DSPE (12.5:37.5:40:4.36:0.64))<br>NGR-SL-S (SPC:CH: PEG-DSPE:NGR-PEG-DSPE (50:40:4.36:0.64))<br>NGR-SL-H (HSPC:CH: PEG-DSPE:NGR-PEG-DSPE (50:40:4.36:0.64))<br>NGR-SL-D (DPPC:CH: PEG-DSPE:NGR-PEG-DSPE (50:40:4.36:0.64))	Extrusion	Fluorescent labeling (DiR dye) of liposomes.	Female BALB/c nude mice, bearing HT1080 xenograft.	i.v.	1.7 mg/kg.	NGR-motif attached to PEGylated liposomes.	20 hours after injection: Signal in tumor highest with NGR-SL-HS liposomes. Uptake in liver, spleen, lung and kidney variable.	[2]
DMPC:DMPG (7:3) liposomes.	Vortexing.	99m$_{TC}$ labelling	Human patients.	i.v.	Lipid dose of 150, 300 or 450 mg/m^2 of body surface area.	n.a.	Liver, spleen and lungs.	[3]
Liposomes<br>A. POPC:Cholesterol (55:45);<br>B. DSPC:DSPG:Cholesterol (53:21:26);<br>C. DOTAP:DOPC (51.5:48.5).	Extrusion	Bilayer labelling with rhodamine PE	Fluorescently-labelled zebrafish embryos.	i.v.	1 nL of 1 mM total lipids.	Zeta Potential<br>A. -15.8;<br>B. -33.7;<br>C. +46.0 mV.	1 hour after injection: intensity in circulation: A>B>C. Differences at tissue level.	[4]
POPC:Cholesterol (55:45).	Extrusion	Bilayer labelling with rhodamine PE	Fluorescently-labelled zebrafish embryos.	i.v.	1 nL of 1 mM total lipids.	Size:<br>114.5 - 122.1 nm<br>325.4 nm<br>464.5 nm	Enhanced uptake by macrophages.	
POPC:Cholesterol:DOPE-mPEG2000 (50:41:9).	Extrusion	Bilayer labelling with rhodamine PE	Fluorescently-labelled zebrafish embryos.	i.v.	1 nL of 1 mM total lipids.	Surface PEGylation	Inhibited phagocytotic uptake.	
DOPC<br>DSPC<br>DOPG	Extrusion	Bilayer labelling with rhodamine PE	Fluorescently-labelled zebrafish	i.v.	1 nL of 1 mM total lipids.	Zeta Potential<br>-11.3; -3.4; -37.1; -45.9; +35.6; -17.2 mV.	Differential distribution of liposome types over blood vessel<br>Network.	

DSPG<br>DOTAP<br>POPC			embryos.					
DOPG	Extrusion	Bilayer labelling with rhodamine PE	Tg(TIE2GFP) 287Sato/J mice	r.o.	100 µL of 10 mM.	n.a.	1 hour after injection: clearance from circulation, accumulation in liver.	
PC:Cholesterol (55:45)	Extrusion	Fluorescent labeling (Cy5.5-NHS dye).	6–8 weeks old SKH1-hr hairless mice	i.v. and inhalation.	n.r.	n.a.	24 hours after administration:<br>i.v.: Kidney 41%; Liver 39%; Spleen 10%; Lungs 6%; Heart 4%.<br>inhalation: Lungs 80%; Kidney 9%; Liver 7%; Heart 2%; Spleen 1%; Brain 1%.	[5]
DPPC : cholesterol : DSPE-PEG2000 in mole ratios of 80 : 0 : 5, 80 : 10 : 5, 80 : 20 : 5, and 80 : 40 : 5.	Sonication.	Fluorescent labeling (DiR dye) of liposomes.	Male Kun Ming mice.	inhalation.	100 µL	Varying cholesterol content.	0.5, 2, 4, 6, 8, 12, 24 and 48 h after administration:<br>Only signal in lungs observed, no significant differences between formulations.	[6]
PG : PC : Chol<br>PI : PC : Chol<br>Sulf : PC : Chol<br>GM1: PC : Chol	Extrusion.	Radioactive labeling (deferoxamine-^{67}Gallium).	Female Swiss Webster mice.	i.v.	1 µmol phospholipid per mouse	Varying formulation.	4 hours after injection:<br>PG : PC : Chol – Liver and Spleen: 71 .5%, Carcass and Skin: 21.5%, Blood: 5.8%, Rest (incl. kidneys, gut, lungs and heart): 1.2%<br>PI : PC : Chol – Liver and Spleen: 37.6%, Carcass and Skin: 25.3%, Blood: 29.4%, Rest: 7.8%<br>Sulf : PC : Chol – Liver and Spleen: 32.7%, Carcass and Skin: 30.3%, Blood: 33.6%, Rest: 3.4%<br>GM1 : PC : Chol – Liver and Spleen: 34.0%, Carcass and Skin: 21.2%, Blood: 33.3%, Rest: 11.5%	[7]
Commercial liposomes, undisclosed formulation.<br>EO771-, 4T1-, or 67NR-cell derived EVs.	-<br>UC, UF and SEC.	Fluorescent labeling (DiD dye) of liposomes and EVs.	Female C57BL/6 and BALB/c mice aged 8 to 12 weeks.	i.v.	Unclear what dose of liposomes was used.<br>EVs:<br>EO771: 1.6 x 10^{11} particles.<br>4T1 and 67NR: 1.2 x 10^{11} particles.	Comparison liposomes versus EVs from different sources.	24 hours after injection:<br>Liposomes: Liver or Liver~Kidney (distribution varies between experiments).<br>EO771: Liver > Spleen.<br>4T1: Lung > Liver > Kidney<br>67NR: Lung ~ Liver<br>*Low radiance for liposomes in comparison with EVs. Dose liposomes administered not given. Equal fluorescence per particle between EVs from different source cells and the liposomes used, was not reported.	[8]
Human embryonic kidney Expi293F cell derived EVs	UC + optiprep-based density separation.	Fluorescent labeling (DiR and mCherry),	Female BALB/c mice aged 6−8 weeks, CT26	i.v.	1 × 10^{11} EVs per animal in 100 µL.	Comparison between various labelling methods.	24 hours after injection:<br>DiR - Liver and spleen main sites of accumulation, minor signal in lungs.<br>mCherry - Signal not above PBS control.	[9]

		Radioactive (^{111}In) labeling, Luminescent labeling (NanoLuc) of EVs.	tumor-bearing.				^{111}In - Liver > Spleen > Kidney.<br>NanoLuc - Lungs main site of accumulation.	
Normal human foreskin fibroblast derived EVs.	UC	Fluorescent labeling (PKH67 dye) of EVs.	Adult C57BL/6 mice.	i.p.	10^8 EVs.	n.a.	24 hours after injection:<br>Liver > Lung > Pancreas > Brain > Spleen > Kidney > GI tract.	[10]
4T1 cell derived EVs, vesicles from 4T1 EV lipid extracts, PC:Chol liposomes.	UC + sucrose-based density separation.	Fluorescent labeling (DiR dye) of EVs and liposomes.	4-week old Balb/c mice, with 4T1 cells inoculated in mammary fat pad.	i.v.	60 µg.	Comparison between particles of different origin.	1, 8 and 24 hours after injection:<br>liver > spleen, limited uptake in lungs and kidneys, no accumulation in tumour tissue.	[11]
PC3 and MCF7 EVs, PC:Chol liposomes.	UC + sucrose-based density separation.	Radioactive (^{111}In) labeling.	4-week old athymic nude (NU/J) mice, also with PC3 cells inoculated subcutaneously in the flank.	i.v.	60 µg.	Comparison between particles of different origin.	24 hours after injection:<br><br>PC3 EVs and PC:Chol liposomes: Liver > spleen > kidneys<br>MCF7 EVs: Spleen > liver > kidneys<br><br>Little accumulation in tumour tissue. Presence of tumour tissue had no influence on biodistribution.	
4T1 cell derived EVs.	UC + sucrose-based density separation.	Fluorescent labeling (DiR dye) of EVs and liposomes.	4-week old Balb/c, athymic nude (NU/J), and NOD.CB17-Prkdcscid/J mice, with 4T1 cells inoculated in mammary fat pad.	i.v.	60 µg.	Different mouse models explored.	20 mins and 2 hours after injection:<br>Liver main site in all mouse models.<br>Slower uptake of EVs in mice with impaired innate immune system and a complement deficiency.	
4T1 cell derived EVs.	UC + sucrose-based density separation.	Fluorescent labeling (DiR dye) of EVs and liposomes.	4-week old Balb/c mice.	i.v.	400 µg.	High dose.	Death of mouse 3 minutes after injection. Main site of accumulation: lungs.	

4T1 cell derived EVs, PC:Chol liposomes.	UC + sucrose-based density separation.	Fluorescent labeling (DiR dye) of EVs and liposomes.	4-week old Balb/c mice, with 4T1 cells inoculated in mammary fat pad.	Intratumoural.	60 µg.	Different site of injection.	1, 12 and 24 hours after injection:<br>Tumour main site of accumulation.	
U937 cell derived EVs and CDNs	UC	Fluorescent labeling (Cy7-NHS dye) of EVs and CDNs.	5-week old white BALB/c mice, also CT26 mouse colon adenocarcinoma bearing.	i.v.	40 µg.	n.a.	24 hours after injection:<br>CDNs in non-tumour mice: Liver > brain > kidney > colon.<br>CDNs in tumour bearing mice: Liver > kidney > tumour ~ colon.<br>EVs in tumour bearing mice: Liver > kidney > tumour.<br>CDNs: Higher fluorescence levels overall than EVs.	[12]
Raw264.7 CDNs	Extrusion and UC	Fluorescent labeling (Cy7-NHS dye) of CDNs.	5-week old male BALB/c mice, CT26 mouse colon adenocarcinoma bearing.	i.v.	50 µg of total protein	n.a.	12 hours after injection:<br>CDNs in non-tumor mice: Liver > lung ~ spleen > kidney.<br>CDNs in tumor bearing mice: Tumor ~ liver ~ spleen ~ lung > kidney.	[13]
HEK293T cell derived EVs	UC	Fluorescent labelling (DiR dye) of EVs.	female NMRI mice	i.v.	$1.0x10^{10}$ particles/gram body weight	n.a.	24 hours after injection: highest EV accumulation in liver, less in spleen, gastrointestinal tract and lungs.	[14]
HEK293T cell derived EVs	UC	Fluorescent labelling (CD63-EGFP fusion protein) of EVs.	female NMRI mice	i.v.	$1.0x10^{10}$ particles/gram body weight	n.a.	24 hours after injection: EGFP-positive EVs detected in liver and spleen parenchyma, negligible EGFP-levels detected in lungs and kidneys	
HEK293T cell derived EVs	UC	Fluorescent labelling (DiR dye) of EVs.	female NMRI mice	i.v.	$1.5x10^{10}$, $1.0x10^{10}$ and $0.25x10^{10}$ particles/gram body weight	Different quantities of EVs administered.	EV accumulation mainly in liver. Spleen, gastrointestinal tract and lungs secondary sites. Dose can shift relative distribution among organs. Liver: decrease with increasing dose; Spleen: no difference; Gastrointestinal tract and lungs: increase with increasing dose.	

HEK293T cell derived EVs	UC	Fluorescent labelling (DiR dye) of EVs.	female NMRI mice	i.v.<br>i.p.<br>s.c.	1.0x10^{10} particles/gram body weight	Different sites of injection.	i.v. injection - main site: liver, secondary sites: spleen, gastrointestinal tract and lungs.<br>i.p. injection - main site: liver and gastrointestinal tract, secondary site: pancreas.<br>s.c. injection - main site: GI tract, secondary sites: liver, pancreas and lungs.<br>i.p. and s.c. injection: lower EV accumulation in liver and spleen, increased accumulation in pancreas and GI tract.<br>i.p. injection total fluorescence somewhat enhanced, s.c. injection reduced compared to i.v.	
EVs from: C2C12 mouse muscle cells, B16-F10 mouse melanoma cells, mouse dendritic cells; OLN-93 rat oligodendrocytes, HEK293T cells, primary human mesenchymal stem cells.	UC	Fluorescent labelling (DiR dye) of EVs.	female NMRI mice	i.v.	1.0x10^{10} particles/gram body weight	Cross-species comparison for intrinsic tropism.	EVs from mouse origin accumulated in liver, spleen, GI-tract and lungs.<br>Liver: C2C12 > B16F10 > DC-derived EVs.<br>Lung: B16F10-EVs > DC-EVs > C2C12 EVs.<br>GI-tract: B16F10 EVs > C2C12-EVs > DC-EVs.<br>Spleen: DC-EVs > C2C12-EVs and B16F10-EVs.<br>EVs from other species had a similar biodistribution profile.<br>Liver: MSC-EVs > OLN93-EVs and HEK293T-EVs.<br>GI-tract: OLN93-EVs > HEK293T-EVs > MSC-EVs.	
HEK293T cell derived EVs	UC	Fluorescent labelling (DiR dye) of EVs.	B16-F10-mouse melanoma tumour bearing female C57BL/6 mice	i.v.	1.0x10^{10} particles/gram body weight		24 hours after injection: highest EV accumulation in liver, less in spleen, gastrointestinal tract and lungs. Tumour tissue a very minor site in comparison (3% of total tissue fluorescence).	
HEK293T cell derived EVs	UC	Fluorescent labelling (DiR dye) of EVs.	female C57BL/6 mice	i.v.	1.0x10^{10} particles/gram body weight	Labelling of EVs with RVG-peptide, by fusion to the EV-tropic protein LAMP2b.	24 hours after injection: highest EV accumulation in liver, less in spleen, gastrointestinal tract and lungs. RVG-EVs had significantly increased signal in brain, heart and higher though not significant signal in muscle, compared to non-RVG-EVs.	
EL-4 - mouse lymphoma cell line derived EVs	UC	Fluorescent labelling (IRDye 800 dye) of EVs.	female C57BL/6j mice	i.p.	Not traceable to EV dose.	n.a.	1 hour after injection: liver, lung, kidney, and spleen	[15]

Mainly EL-4 - mouse lymphoma cell line derived EVs, also 3T3L1, 4T1, CT26 and A20 EVs; CT26 and A20 microparticles.	UC for enrichment of exosomes (30-100 nm) and larger microparticles (500 nm-1 m)	Fluorescent labelling (IRDye 800 dye or DiR dye) of EVs.	C57BL/6j mice	i.n.	10 µg (protein).	n.a.	30 mins after delivery: brain – olfactory bulb mainly (exosomes); no microparticles observed. 3 hours after delivery: brain and intestine (exosomes); intestine and lung (microparticles).	[16]
B16BL6 murine melanoma cell line derived EVs.	UC	Lactadherin and Gaussia luciferase fusion protein.	Five-week-old male BALB/c and C57BL/6 mice.	i.v.	5 µg.	n.a.	10, 30 and 60 min after injection: liver > lung > spleen > kidney. 4 hours after injection: lung > spleen.	[17]
B16BL6 murine melanoma cell line derived EVs.	UC	Lactadherin and Gaussia luciferase fusion protein.	Five-week-old male BALB/c mice.	i.v.	5 µg.	n.a.	10, 30, 60 and 240 min after injection: main sites liver, spleen, lung.	[18]
						Clodronate liposome-treated mice, to deplete macrophages.	10, 30, 60 and 240 min after injection: signal in liver, spleen, lung increased compared to control: decreased clearance.	
B16BL6 murine melanoma cell line derived EVs.	UC	Radioactive labeling.	Five-week-old male BALB/c mice.	i.v.	4 µg.	n.a.	5 min after injection: Main site of distribution is the liver, minor sites are the lungs.	[19]
						Pre-administration of 0.8 mg phosphatidylserine- or phosphatidylglycerol-rich liposomes 2 min before injection of EVs.	Clearance of EVs from blood reduced with pre-administration of liposomes. EV accumulation in liver diminished.	
B16-F10 murine melanoma cell line derived EVs.	UC and AF4	Fluorescent labeling (NIR dye) of EVs.	6-week-old female C57BL/6 mice	r.o.	10 µg.	n.a.	24 hours after injection: EVs accumulated mainly in liver (~84% of total signal), followed by spleen (~14%), bone marrow (~1.6%), lungs (~0.23%), lymph nodes (~0.07%) and kidneys (~0.08%).	[20]

B16-F10 murine melanoma cell line derived EVs.	UC	Fluorescent labelling (PKH67 dye) of EVs.	8- to 10-week-old C57BL/6J female mice	i.v.	5-10 µg.	n.a.	5 min after injection: EVs detected in blood vessels of organs.<br>24 hours after injection: EVs found in lung, bone marrow, liver and spleen, but absent from circulation.	[21]
Mesenchymal stem cell EVs.	UC	Fluorescent labelling (DiD and DiL dye) of EVs and EVs derived from DiD/DiL-labelled cells.	6- to 8-week-old CD1 male nude mice, including an AKI model induced by intramuscular glycerol injection.	i.v.	200 µg.	n.a.	5 and 24 hours after injection: liver > spleen > lung as major sites, signal in AKI model enhanced overall.	[22]
HEK293T EVs.	UC	Genetic labelling of parent cells with *Gaussia* luciferase.	6-week-old athymic nude mice.	r.o.	100 µg.	n.a.	1 hour after injection: main site of accumulation is liver, followed by spleen.	[23]
			6-week-old athymic nude mice xenografted with Gli36 tumours on left and right chest regions.	i.v.	100 µg.	n.a.	1 hour after injection: liver, spleen and tumour main sites of accumulation.	
Mouse B16BL6 melanoma cell, C2C12 myoblast cell, NIH3T3 fibroblast, MAEC aortic endothelial cell, and RAW264.7 macrophage-like cell EVs.	UC	Lactadherin and Gaussia luciferase fusion protein.	Five-week-old male BALB/c mice.	i.v.	5 µg.	n.a.	EVs from all cell types ~100 nm diameter; negative zeta potential of ~−40 mV. 5 min after injection, all accumulated mainly in the liver.	[24]
Outer membrane vesicles from bacterial origin (*Escherichia coli*).	UC	Fluorescent labelling (Cy7-NHS dye) of EVs.	SKH1-E hairless mice.	i.p.	15 µg.	n.a.	Imaging: 3 hours after injection: liver > lung > spleen - kidney as major sites. 24 hours: liver.<br>ELISA-based analysis: liver > lung > spleen > kidney at 3, 6, 12 and 24 hours.	[25]

Abbreviations: AF4 = asymmetric flow field-flow fractionation; AKI = acute kidney injury; i.n. = intranasal; i.p. intraperitoneal; i.v. = intravenous; s.c. = subcutaneous; SEC = Size Exclusion Chromatography; r.o. = retro-orbital; UC = ultracentrifugation; UF = ultrafiltration.

References

[1] B. Dos Santos Rodrigues, S. Arora, T. Kanekiyo, J. Singh, *Brain Res* **2020**, 1734, 146738.
[2] J. Chen, A. Lin, P. Peng, Y. Wang, W. Gu, Y. Liu, *Drug Deliv* **2016**, 23, 1426.
[3] G. Lopez-Berestein, L. Kasi, M. G. Rosenblum, T. Haynie, M. Jahns, H. Glenn, R. Mehta, G. M. Mavligit, E. M. Hersh, *Cancer Res* **1984**, 44, 375.
[4] F. Campbell, F. L. Bos, S. Sieber, G. Arias-Alpizar, B. E. Koch, J. Huwyler, A. Kros, J. Bussmann, *ACS Nano* **2018**, 12, 2138.
[5] V. Ivanova, O. B. Garbuzenko, K. R. Reuhl, D. C. Reimer, V. P. Pozharov, T. Minko, *Eur J Pharm Biopharm* **2013**, 84, 335.
[6] J. Zhao, J. Su, L. Qin, X. Zhang, S. Mao, *Biomater Sci* **2020**, 8, 6786.
[7] D. Papahadjopoulos, A. Gabizon, *Ann N Y Acad Sci* **1987**, 507, 64.
[8] S. W. Wen, J. Sceneay, L. G. Lima, C. S. Wong, M. Becker, S. Krumeich, R. J. Lobb, V. Castillo, K. N. Wong, S. Ellis, B. S. Parker, A. Moller, *Cancer Res* **2016**, 76, 6816.
[9] E. Lazaro-Ibanez, F. N. Faruqu, A. F. Saleh, A. M. Silva, J. Tzu-Wen Wang, J. Rak, K. T. Al-Jamal, N. Dekker, *ACS Nano* **2021**, 15, 3212.
[10] S. Kamerkar, V. S. LeBleu, H. Sugimoto, S. Yang, C. F. Ruivo, S. A. Melo, J. J. Lee, R. Kalluri, *Nature* **2017**, 546, 498.
[11] T. Smyth, M. Kullberg, N. Malik, P. Smith-Jones, M. W. Graner, T. J. Anchordoquy, *J Control Release* **2015**, 199, 145.
[12] W. J. Goh, S. Zou, W. Y. Ong, F. Torta, A. F. Alexandra, R. M. Schiffelers, G. Storm, J. W. Wang, B. Czarny, G. Pastorin, *Sci Rep* **2017**, 7, 14322.
[13] S. C. Jang, O. Y. Kim, C. M. Yoon, D. S. Choi, T. Y. Roh, J. Park, J. Nilsson, J. Lotvall, Y. K. Kim, Y. S. Gho, *ACS Nano* **2013**, 7, 7698.
[14] O. P. Wiklander, J. Z. Nordin, A. O'Loughlin, Y. Gustafsson, G. Corso, I. Mager, P. Vader, Y. Lee, H. Sork, Y. Seow, N. Heldring, L. Alvarez-Erviti, C. I. Smith, K. Le Blanc, P. Macchiarini, P. Jungebluth, M. J. Wood, S. E. Andaloussi, *J Extracell Vesicles* **2015**, 4, 26316.
[15] D. Sun, X. Zhuang, X. Xiang, Y. Liu, S. Zhang, C. Liu, S. Barnes, W. Grizzle, D. Miller, H. G. Zhang, *Mol Ther* **2010**, 18, 1606.
[16] X. Zhuang, X. Xiang, W. Grizzle, D. Sun, S. Zhang, R. C. Axtell, S. Ju, J. Mu, L. Zhang, L. Steinman, D. Miller, H. G. Zhang, *Mol Ther* **2011**, 19, 1769.
[17] Y. Takahashi, M. Nishikawa, H. Shinotsuka, Y. Matsui, S. Ohara, T. Imai, Y. Takakura, *J Biotechnol* **2013**, 165, 77.
[18] T. Imai, Y. Takahashi, M. Nishikawa, K. Kato, M. Morishita, T. Yamashita, A. Matsumoto, C. Charoenviriyakul, Y. Takakura, *J Extracell Vesicles* **2015**, 4, 26238.
[19] A. Matsumoto, Y. Takahashi, M. Nishikawa, K. Sano, M. Morishita, C. Charoenviriyakul, H. Saji, Y. Takakura, *J Pharm Sci* **2017**, 106, 168.
[20] H. Zhang, D. Freitas, H. S. Kim, K. Fabijanic, Z. Li, H. Chen, M. T. Mark, H. Molina, A. B. Martin, L. Bojmar, J. Fang, S. Rampersaud, A. Hoshino, I. Matei, C. M. Kenific, M. Nakajima, A. P. Mutvei, P. Sansone, W. Buehring, H. Wang, J. P. Jimenez, L. Cohen-Gould, N. Paknejad, M. Brendel, K. Manova-Todorova, A. Magalhaes, J. A. Ferreira, H. Osorio, A. M. Silva, A. Massey, J. R. Cubillos-Ruiz, G. Galletti, P. Giannakakou, A. M. Cuervo, J. Blenis, R. Schwartz, M. S. Brady, H. Peinado, J. Bromberg, H. Matsui, C. A. Reis, D. Lyden, *Nat Cell Biol* **2018**, 20, 332.
[21] H. Peinado, M. Aleckovic, S. Lavotshkin, I. Matei, B. Costa-Silva, G. Moreno-Bueno, M. Hergueta-Redondo, C. Williams, G. Garcia-Santos, C. Ghajar, A. Nitadori-Hoshino, C. Hoffman, K. Badal, B. A. Garcia, M. K. Callahan, J. Yuan, V. R. Martins, J. Skog, R. N. Kaplan, M. S. Brady, J. D. Wolchok, P. B. Chapman, Y. Kang, J. Bromberg, D. Lyden, *Nat Med* **2012**, 18, 883.
[22] C. Grange, M. Tapparo, S. Bruno, D. Chatterjee, P. J. Quesenberry, C. Tetta, G. Camussi, *Int J Mol Med* **2014**, 33, 1055.
[23] C. P. Lai, O. Mardini, M. Ericsson, S. Prabhakar, C. Maguire, J. W. Chen, B. A. Tannous, X. O. Breakefield, *ACS Nano* **2014**, 8, 483.
[24] C. Charoenviriyakul, Y. Takahashi, M. Morishita, A. Matsumoto, M. Nishikawa, Y. Takakura, *Eur J Pharm Sci* **2017**, 96, 316.
[25] S. C. Jang, S. R. Kim, Y. J. Yoon, K. S. Park, J. H. Kim, J. Lee, O. Y. Kim, E. J. Choi, D. K. Kim, D. S. Choi, Y. K. Kim, J. Park, D. Di Vizio, Y. S. Gho, *Small* **2015**, 11, 456.

Supplementary Table 4. Overview of active targeting of EVs.

EV source	Isolation method	Targeting moiety	Modification	Target tissue	Injection	Biodistribution (in vivo)	Results	Ref
	UC	RVG-peptide	TF	Brain in mice	i.v.	n.r. (significant knockdown in GAPDH in brains, not in spleen, liver, and kidneys)	GAPDH siRNA was specifically delivered to neurons, microglia, and oligodendrocytes, resulting in specific gene knockdown.	[1]
CDC	UC	Cardiomyocyte specific peptide	TF	Heart in mice	i.m.	Vast majority in lungs, spleen, and liver.	Increased uptake by cardiomyocytes, decreased cardiomyocyte apoptosis, and higher cardiac retention.	[2]
DC	UC, UF, and DG	αv-integrin-specific iRGD-peptide	TF	MDA-MB-231 mouse tumor	i.v.	Vast majority in liver. Targeting increases tumor accumulation	Inhibition of tumor growth without overt toxicity.	[3]
HEK293 cells	UC	GE11-peptide	TF	EGFR-positive breast cancer xenograft in mice	i.v.	n.r. (targeting increases tumor accumulation)	Significant suppressed tumor growth by delivery of let-7a miRNA.	[4]
HEK293 cells	IK	RVG-peptide	TF	Brain in mice	i.v.	n.r.	Opioid receptor mu (MOR) siRNA delivered by targeted EVs inhibited morphine relapse via downregulation of MOR expression.	[5]
DC	UC	RVG-peptide	TF	Brain in mice	i.v.	n.r.	Alpha-synuclein (α-Syn) siRNA delivered by targeted EVs reduced intraneuronal protein aggregation.	[6]
Neuro2a cells	UC	Anti-EGFR nanobodies	PI	A431 tumor in mice	i.v.	Vast majority in the liver and spleen. Signal in tumor below detection limit.	Functional effects were not studied.	[7]
HEK293 cells	UC	Anti-HER2 scFv antibody	TF	Orthotropic Her2+ BT474 xenografts in mice	i.p.	n.r.	Near-complete growth-arrest of xenografts by HChrR6 mRNA transfer.	[8]
L929 cells	UC	Low-density protein peptide	PI	Glioma in mice	i.v.	Vast majority in the liver, spleen, and kidney. Targeting increases brain accumulation.	Mice treated with targeted EVs showed the longest median survival period.	[9]
BM-MSCs	UC	c(RGDyK) peptide	PI	Ischemic brain in mice	i.v.	Vast majority in the liver. Targeting increases brain accumulation.	Suppression of the inflammatory response and cellular apoptosis in the lesion region.	[10]

Raw264.7 cells	UC and UF	Neuropilin-1-targeted peptide	CC	Glioma in mice	i.v.	Vast majority in liver and spleen. Targeting increases brain accumulation.	Tumors diminished after treatment and survival rate was increased.	[11]
C2C12 cells	UC	M12 muscle targeting-peptide	PI	Muscular dystrophy in mice	i.v.	Vast majority in liver. Targeting increases muscle accumulation	Increase dystrophin expression in muscle by delivery of splice correcting oligomers.	[12]
C2C12 cells	UC	RVG-peptide	PI	Brain in mice	i.v.	Vast majority in liver. Targeting increases brain accumulation	Functional effects were not studied.	[12]
C2C12 cells	UC	SP94-peptide	PI	Hepatocellular tumor in mice	i.v.	Vast majority in liver, spleen, and kidneys. Targeting increases tumor accumulation	Functional effects were not studied.	[12]
K562 cells	MBP	RGD-peptide	PRI	Blood vessels in zebrafish	Injection into embryo	Increased accumulation of EVs in blood vessels	Dose-dependent angiogenesis	[13]
HEK293 cells	UC and TFF	CTP-peptide	TF	Heart in mice	i.v.	Vast majority in liver. Targeting increases heart accumulation.	Functional effects were not studied.	[14]
CDC	UF	Ischemic targeting-peptide	PI	Heart in mice	i.v.	Vast majority in liver and kidneys. Targeting increases heart accumulation.	Functional effects were not studied.	[15]
DC	UC	RVG-peptide	TF	Acetylcholine-receptor-rich organs	i.v.	Vast majority in liver, spleen, lungs, and GI-tract. Targeting increases brain and heart accumulation.	Functional effects were not studied.	[16]
CDC	UF	CHP-peptide	PI	Heart in mice	i.v.	Vast majority in liver, spleen, and kidneys. Targeting increases brain accumulation.	Reduced fibrosis and scar size, and increased cellular proliferation and angiogenesis.	[17]
HEK293 cells	UC	Interleukin-3 fragment	TF	CML-xenograft in mice	i.v.	Vast majority in liver, spleen, and kidneys. Targeting increases tumor accumulation.	Cancer cell growth was inhibited by the delivery of imatinib of BCR-ABL siRNA	[18]
PMN	UC	anti-ROS-CII antibody	PI	Arthritic join in mice	i.v.	Vast majority in liver. Targeting increases arthritic joint accumulation.	Accelerated attenuation of clinical and synovial inflammation by the delivery of viral IL-10 and anti-TNF.	[19]
BM-MSCs	UC	IMT-peptide	CC	Heart in mice	i.v.	Vast majority in liver and kidneys. Targeting increases heart accumulation.	Ischemic cardiac repair by ameliorating cardiomyocyte apoptosis by delivery of miR-125b-5p	[20]

Abbreviations: anti-ROS-CII = antibody against damaged arthritic cartilage; BM-MSCs = bone marrow-derived mesenchymal stromal cells; CC = click chemistry; CDC = cardiosphere-derived cells; CHP = cardiac homing peptide; CML = Chronic Myelogenous Leukemia; DC = dendritic cells; DG = density gradient; GI-tract = gastrointestinal tract; IK = isolation kit; i.m. = intramyocardial; IMT = ischemic myocardium-targeted; i.p. intraperitoneal; i.v. = intravenous; MBP = magnetic biomimetic particles; n.r. = not reported; PI = post-insertion; PMN = human neutrophils; PRI = pre-incubation; TF = transfection; TFF = tangential flow filtration; UC = ultracentrifugation; UF – ultrafiltration.

References

[1] L. Alvarez-Erviti, Y. Seow, H. Yin, C. Betts, S. Lakhal, M. J. Wood, *Nat Biotechnol* **2011**, 29, 341.
[2] K. I. Mentkowski, J. K. Lang, *Sci Rep* **2019**, 9, 10041.
[3] Y. Tian, S. Li, J. Song, T. Ji, M. Zhu, G. J. Anderson, J. Wei, G. Nie, *Biomaterials* **2014**, 35, 2383.
[4] S. Ohno, M. Takanashi, K. Sudo, S. Ueda, A. Ishikawa, N. Matsuyama, K. Fujita, T. Mizutani, T. Ohgi, T. Ochiya, N. Gotoh, M. Kuroda, *Mol Ther* **2013**, 21, 185.
[5] Y. Liu, D. Li, Z. Liu, Y. Zhou, D. Chu, X. Li, X. Jiang, D. Hou, X. Chen, Y. Chen, Z. Yang, L. Jin, W. Jiang, C. Tian, G. Zhou, K. Zen, J. Zhang, Y. Zhang, J. Li, C. Y. Zhang, *Sci Rep* **2015**, 5, 17543.
[6] J. M. Cooper, P. B. Wiklander, J. Z. Nordin, R. Al-Shawi, M. J. Wood, M. Vithlani, A. H. Schapira, J. P. Simons, S. El-Andaloussi, L. Alvarez-Erviti, *Mov Disord* **2014**, 29, 1476.
[7] S. A. A. Kooijmans, L. A. L. Fliervoet, R. van der Meel, M. Fens, H. F. G. Heijnen, P. M. P. van Bergen En Henegouwen, P. Vader, R. M. Schiffelers, *J Control Release* **2016**, 224, 77.
[8] J. H. Wang, A. V. Forterre, J. Zhao, D. O. Frimannsson, A. Delcayre, T. J. Antes, B. Efron, S. S. Jeffrey, M. D. Pegram, A. C. Matin, *Mol Cancer Ther* **2018**, 17, 1133.
[9] Z. Ye, T. Zhang, W. He, H. Jin, C. Liu, Z. Yang, J. Ren, *ACS Appl Mater Interfaces* **2018**, 10, 12341.
[10] T. Tian, H. X. Zhang, C. P. He, S. Fan, Y. L. Zhu, C. Qi, N. P. Huang, Z. D. Xiao, Z. H. Lu, B. A. Tannous, J. Gao, *Biomaterials* **2018**, 150, 137.
[11] G. Jia, Y. Han, Y. An, Y. Ding, C. He, X. Wang, Q. Tang, *Biomaterials* **2018**, 178, 302.
[12] X. Gao, N. Ran, X. Dong, B. Zuo, R. Yang, Q. Zhou, H. M. Moulton, Y. Seow, H. Yin, *Sci Transl Med* **2018**, 10.
[13] J. Wang, W. Li, Z. Lu, L. Zhang, Y. Hu, Q. Li, W. Du, X. Feng, H. Jia, B. F. Liu, *Nanoscale* **2017**, 9, 15598.
[14] H. Kim, N. Yun, D. Mun, J. Y. Kang, S. H. Lee, H. Park, H. Park, B. Joung, *Biochem Biophys Res Commun* **2018**, 499, 803.
[15] T. J. Antes, R. C. Middleton, K. M. Luther, T. Ijichi, K. A. Peck, W. J. Liu, J. Valle, A. K. Echavez, E. Marban, *J Nanobiotechnology* **2018**, 16, 61.
[16] O. P. Wiklander, J. Z. Nordin, A. O'Loughlin, Y. Gustafsson, G. Corso, I. Mager, P. Vader, Y. Lee, H. Sork, Y. Seow, N. Heldring, L. Alvarez-Erviti, C. I. Smith, K. Le Blanc, P. Macchiarini, P. Jungebluth, M. J. Wood, S. E. Andaloussi, *J Extracell Vesicles* **2015**, 4, 26316.
[17] A. Vandergriff, K. Huang, D. Shen, S. Hu, M. T. Hensley, T. G. Caranasos, L. Qian, K. Cheng, *Theranostics* **2018**, 8, 1869.
[18] D. Bellavia, S. Raimondo, G. Calabrese, S. Forte, M. Cristaldi, A. Patinella, L. Memeo, M. Manno, S. Raccosta, P. Diana, G. Cirrincione, G. Giavaresi, F. Monteleone, S. Fontana, G. De Leo, R. Alessandro, *Theranostics* **2017**, 7, 1333.
[19] L. M. Topping, B. L. Thomas, H. I. Rhys, J. L. Tremoleda, M. Foster, M. Seed, M. B. Voisin, C. Vinci, H. L. Law, M. Perretti, L. V. Norling, H. S. Azevedo, A. Nissim, *Front Immunol* **2020**, 11, 10.
[20] L. P. Zhu, T. Tian, J. Y. Wang, J. N. He, T. Chen, M. Pan, L. Xu, H. X. Zhang, X. T. Qiu, C. C. Li, K. K. Wang, H. Shen, G. G. Zhang, Y. P. Bai, *Theranostics* **2018**, 8, 6163.
